# Supplementary figures and images for: Dysregulated heparan sulfate proteoglycan metabolism promotes Ewing sarcoma tumor growth
Source: eLife. 2022 Mar 14;11:e69734. doi: 10.7554/eLife.69734 (PMC8942468; doi:10.7554/eLife.69734)

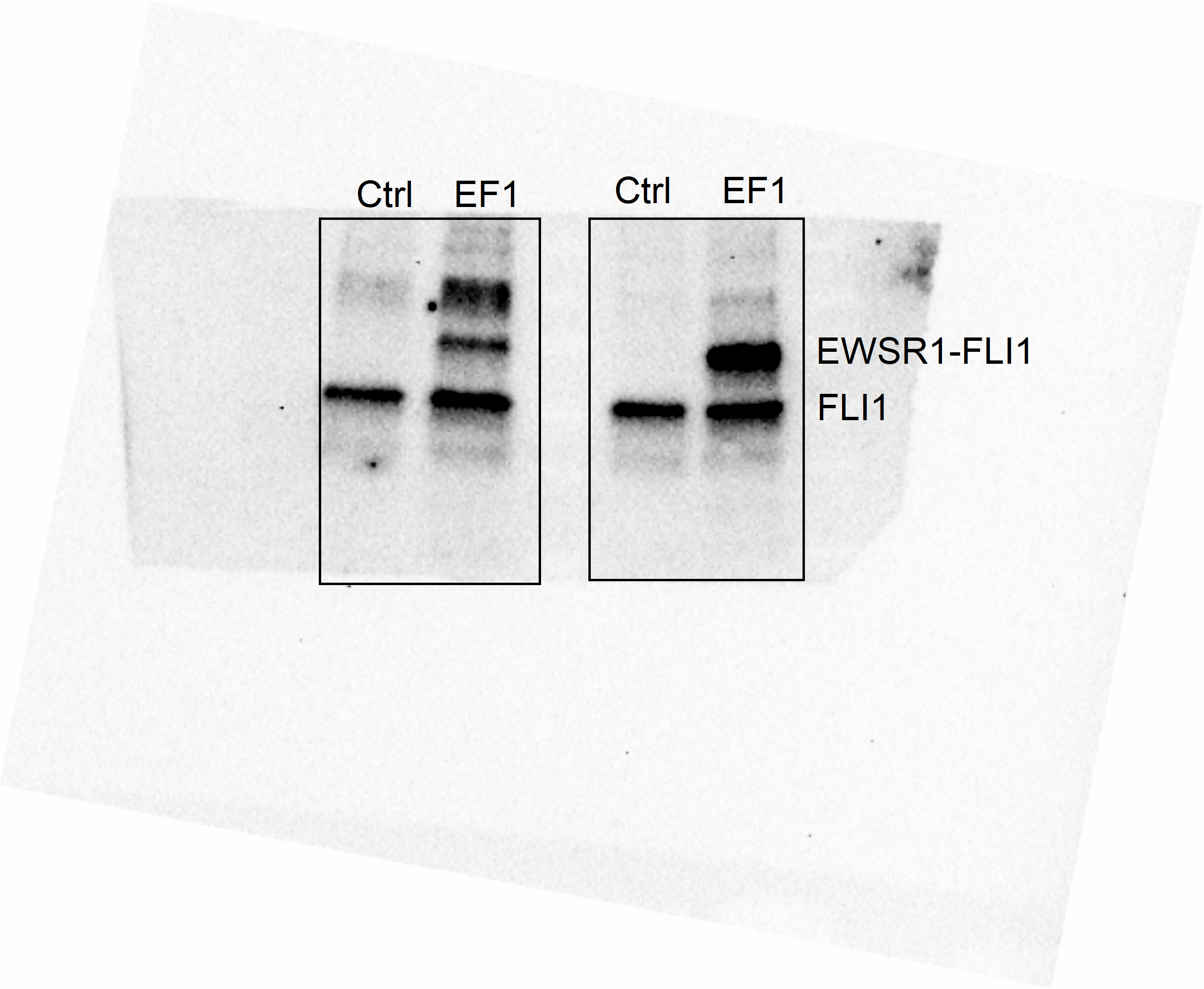

Supplement: Source data 1. [file elife-69734-data1.zip › Vasileva_Source Data/Figure 2-source data 2.tif]

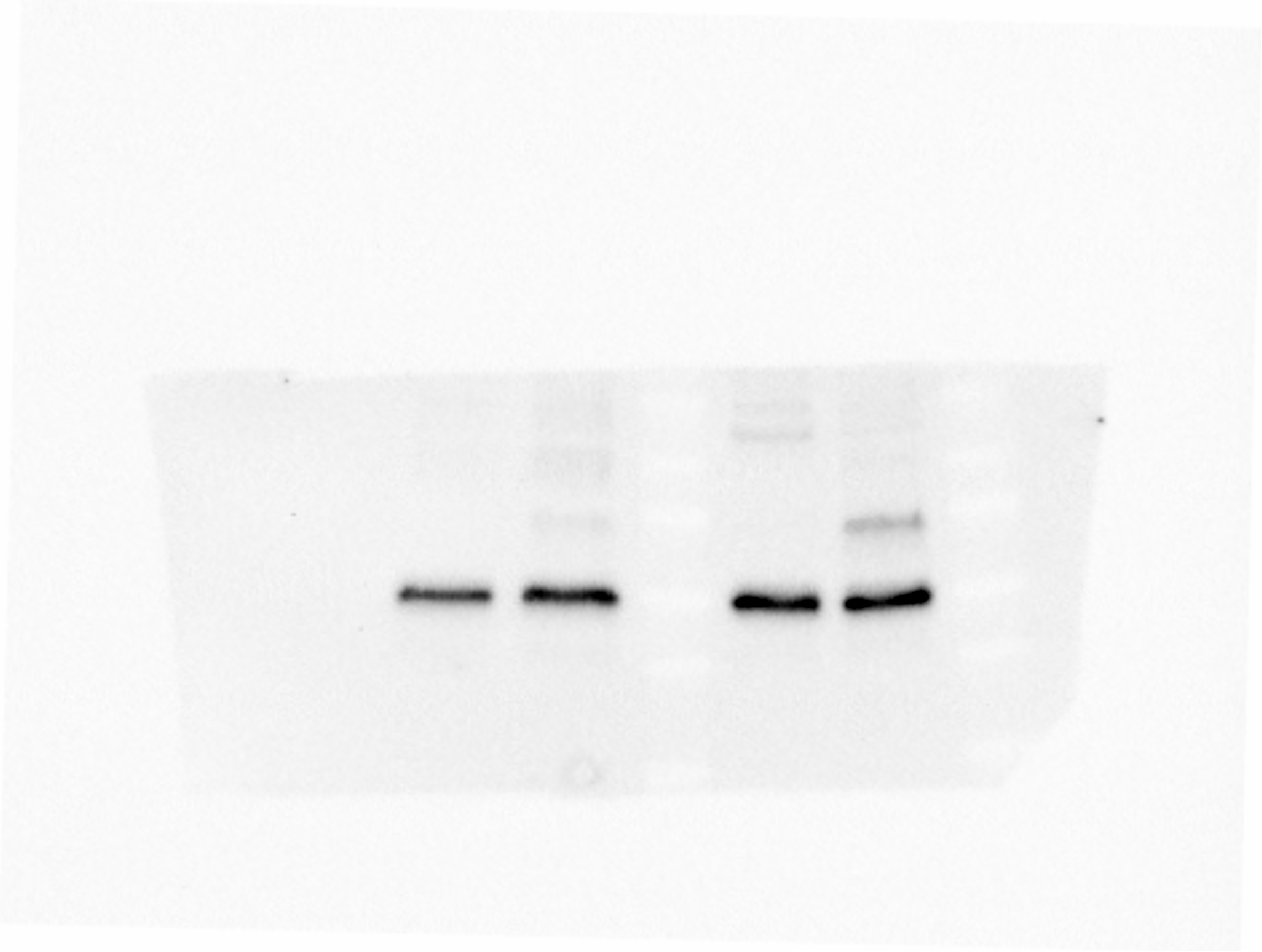

Supplement: Source data 1. [file elife-69734-data1.zip › Vasileva_Source Data/Figure 2-source data 5]

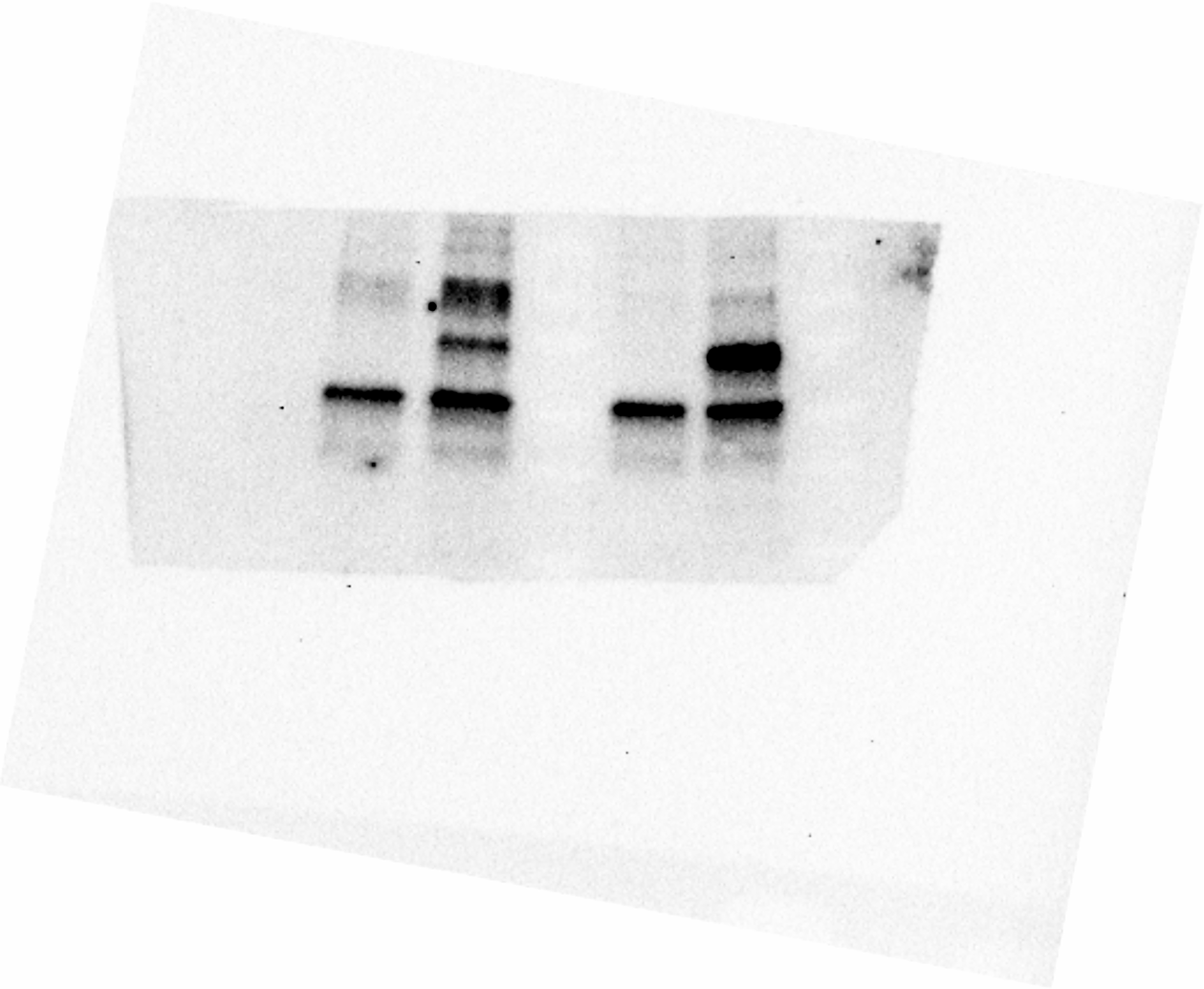

Supplement: Source data 1. [file elife-69734-data1.zip › Vasileva_Source Data/Figure 2-source data 1.tif]

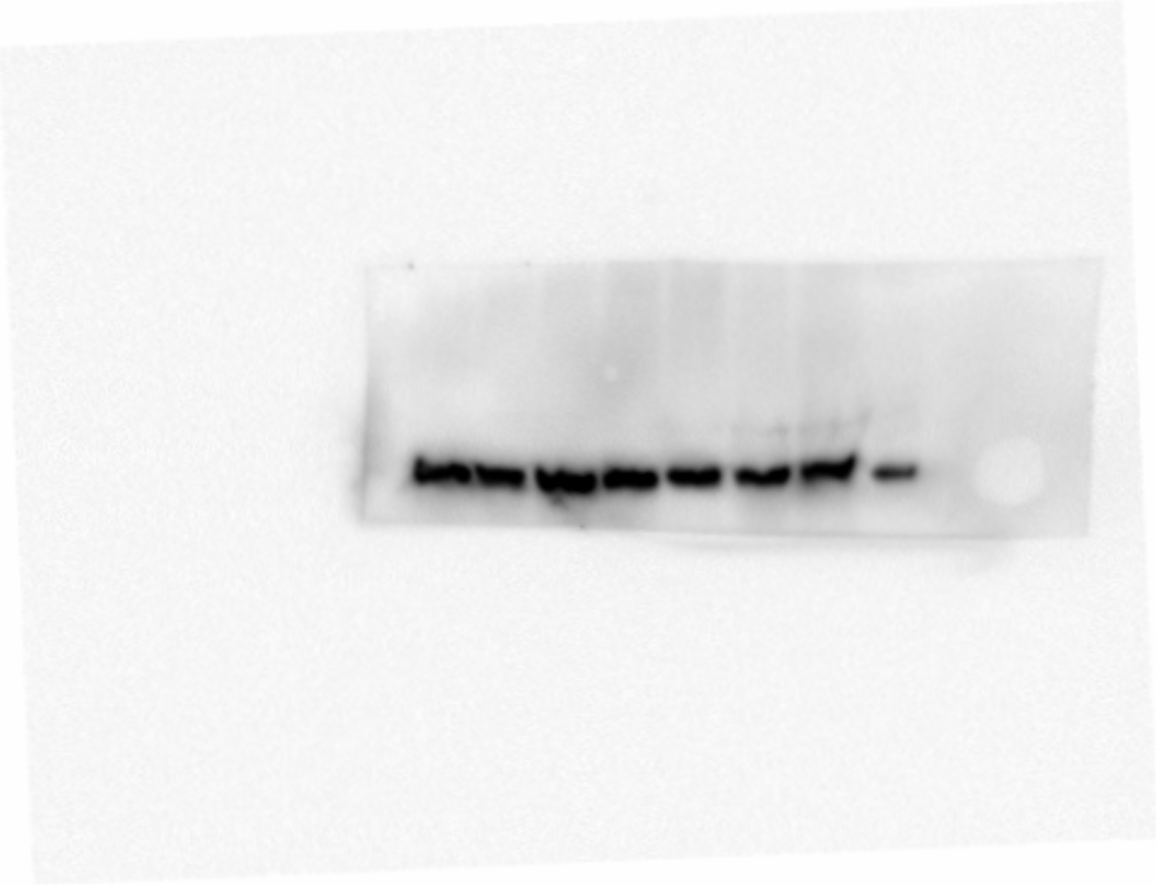

Supplement: Source data 1. [file elife-69734-data1.zip › Vasileva_Source Data/Figure 3-source data 3.tif]

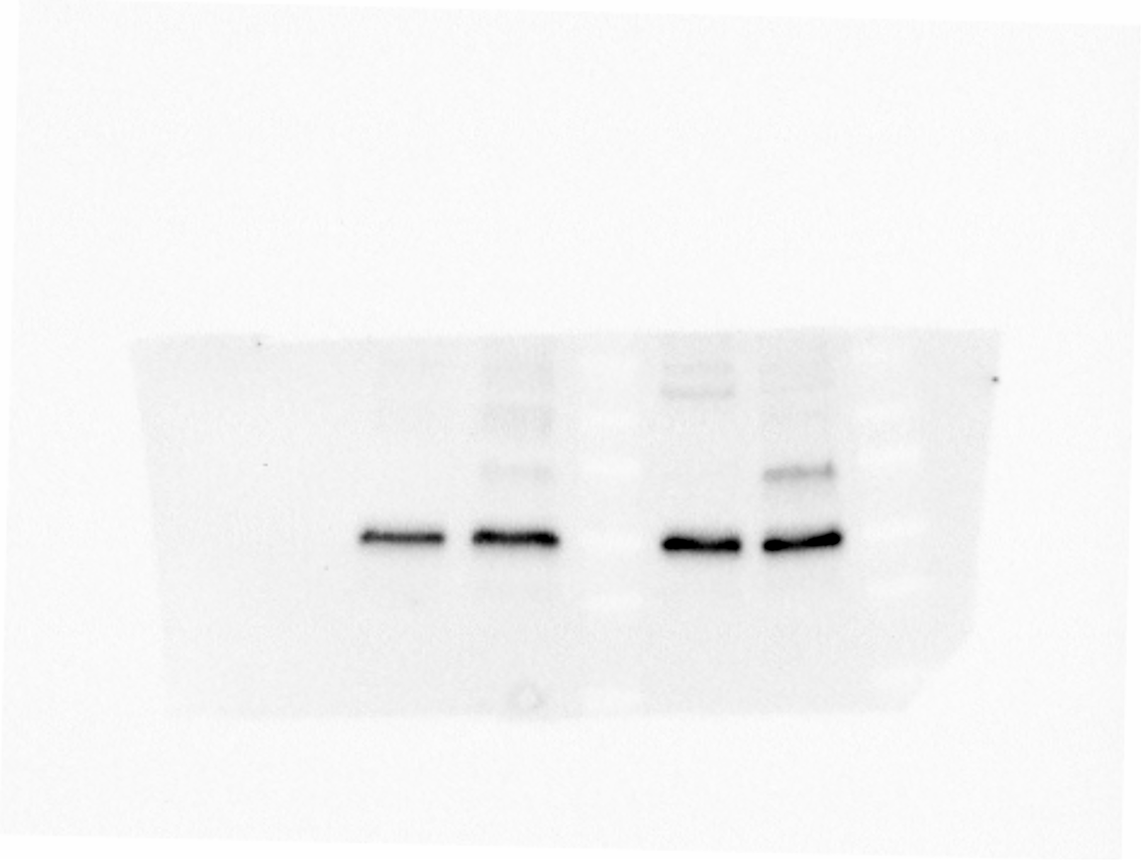

Supplement: Source data 1. [file elife-69734-data1.zip › Vasileva_Source Data/Figure 2-source data 5.tif]

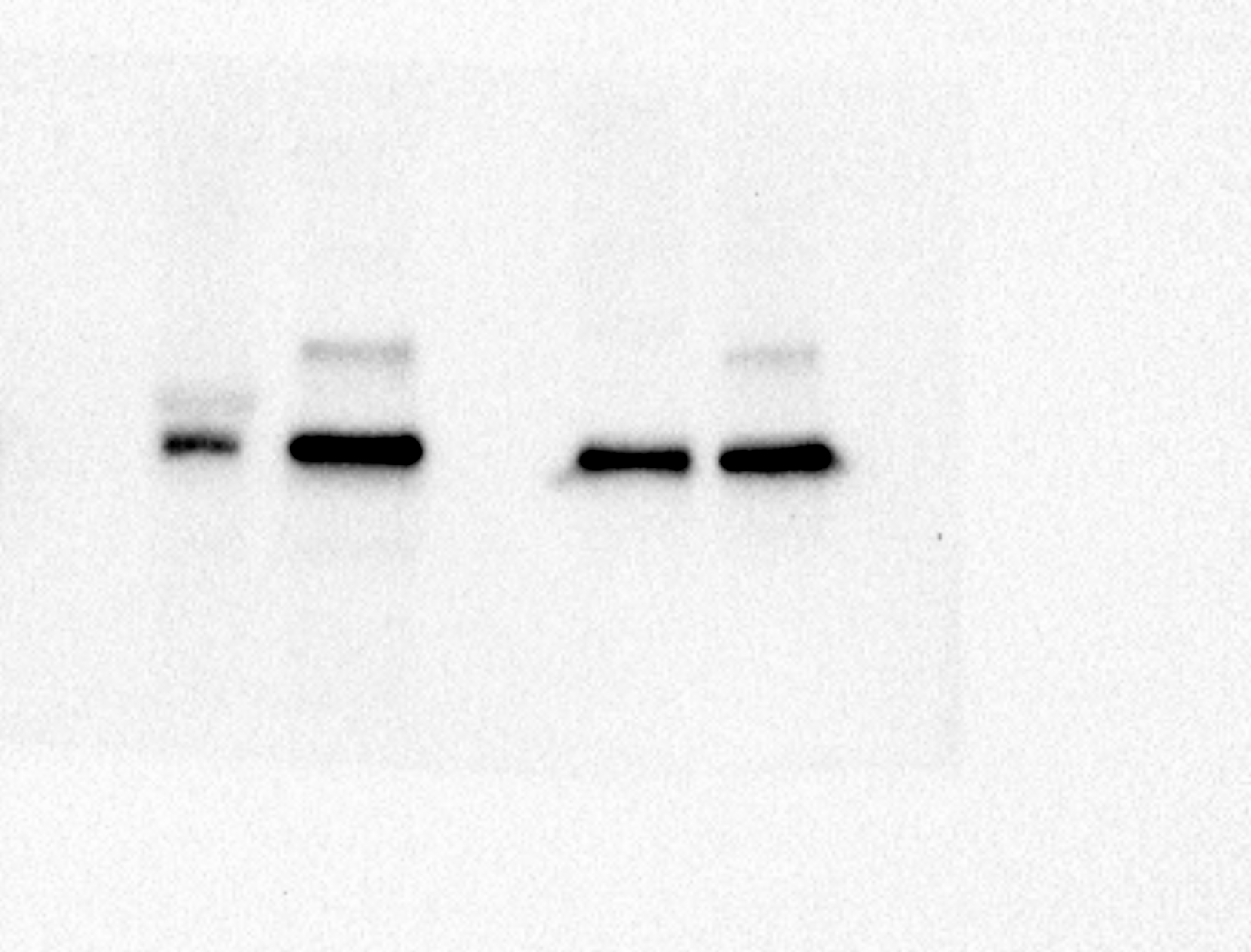

Supplement: Source data 1. [file elife-69734-data1.zip › Vasileva_Source Data/Figure 2-source data 7.tif]

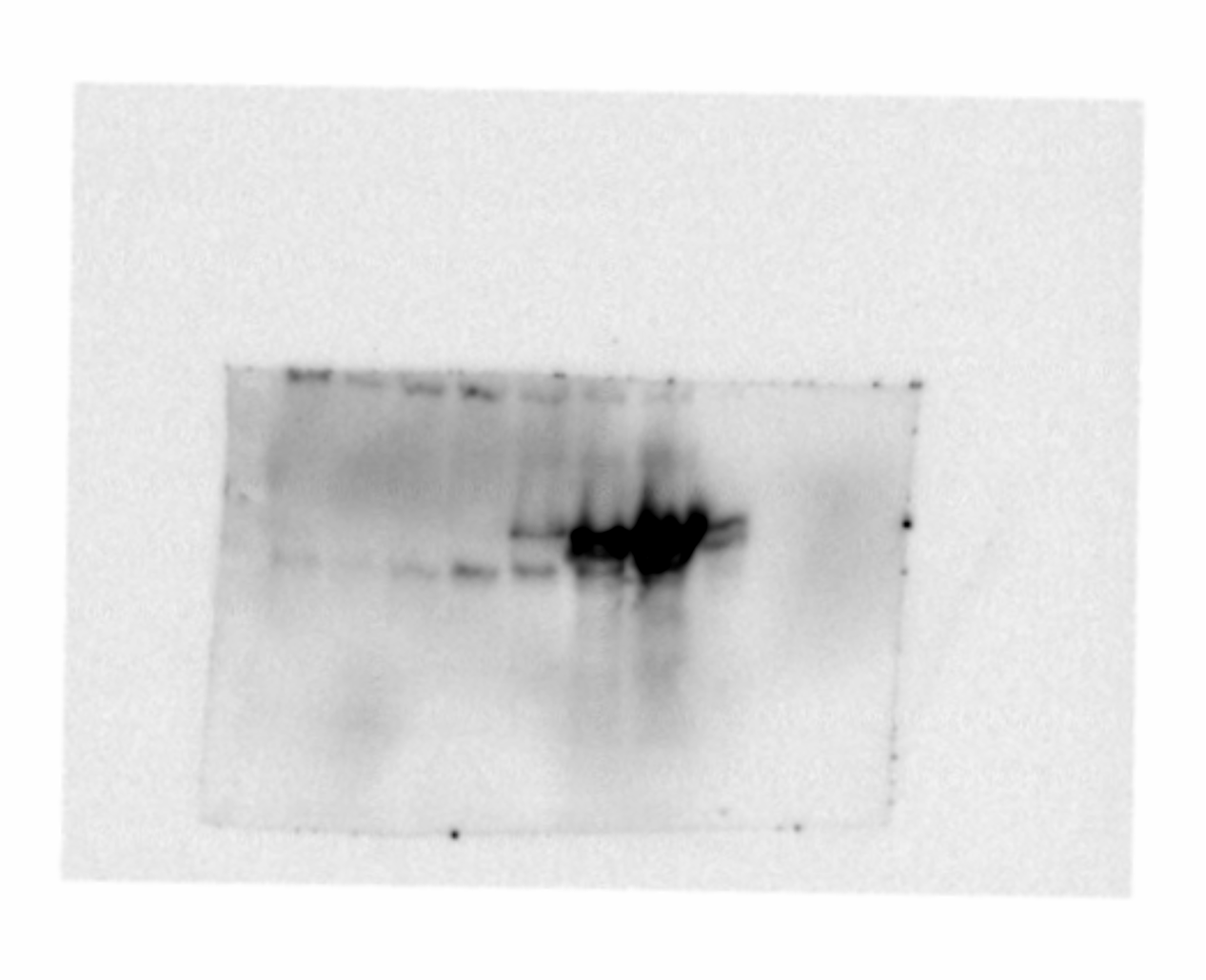

Supplement: Source data 1. [file elife-69734-data1.zip › Vasileva_Source Data/Figure 3-source data 1.tif]

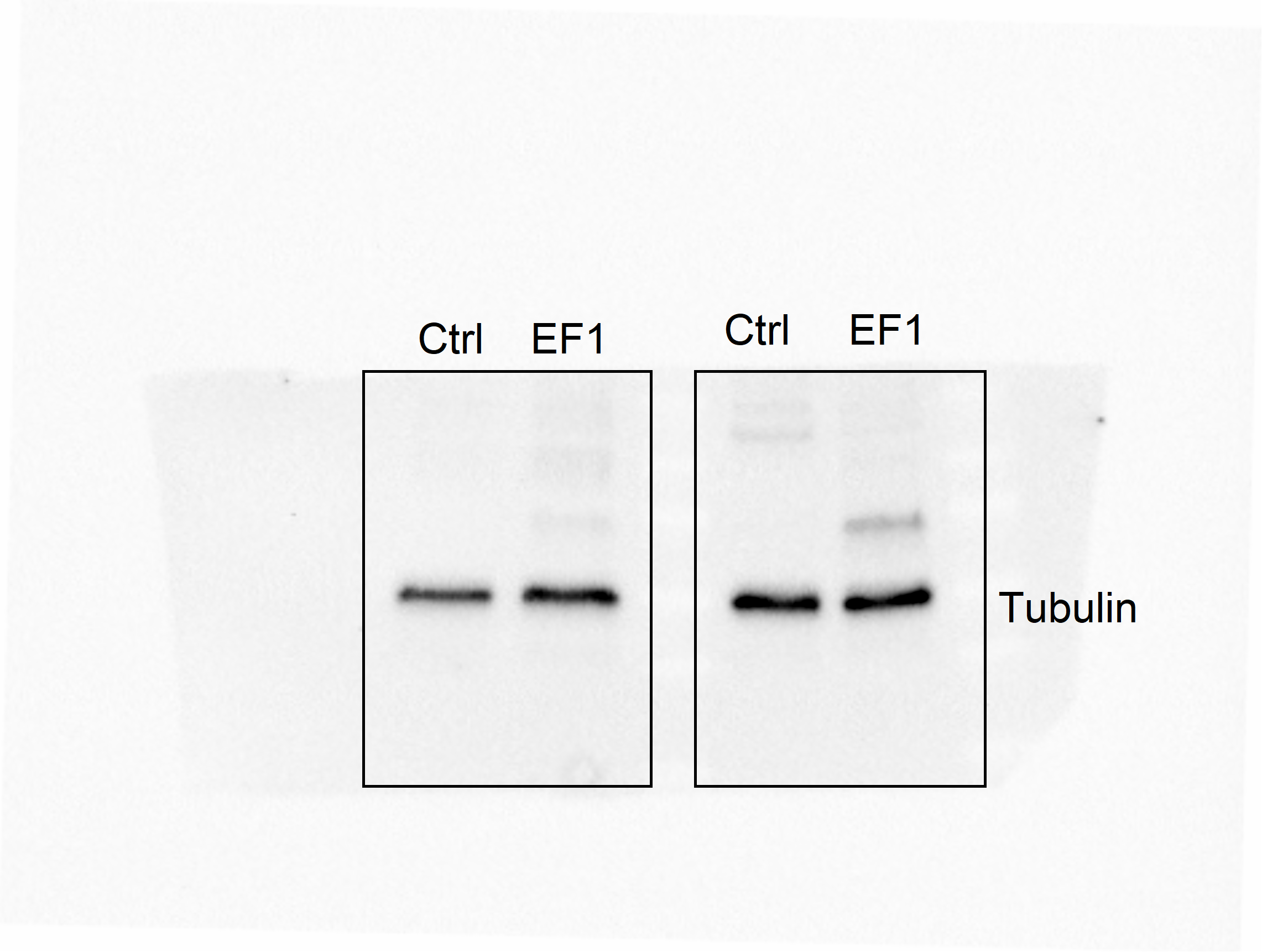

Supplement: Source data 1. [file elife-69734-data1.zip › Vasileva_Source Data/Figure 2-source data 6.tif]

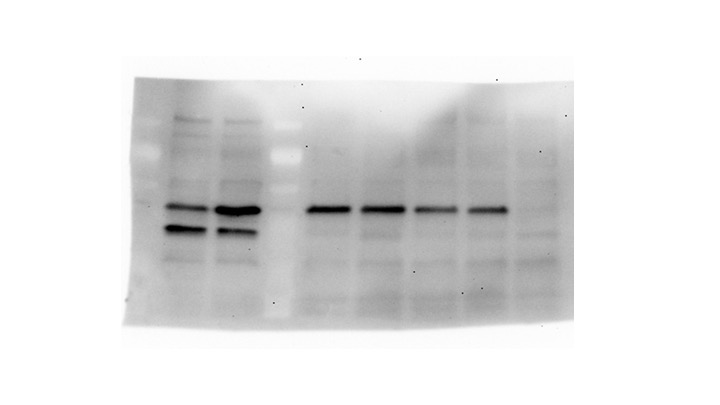

Supplement: Source data 1. [file elife-69734-data1.zip › Vasileva_Source Data/Figure 7-figure supplement 1-source data 1.jpeg]

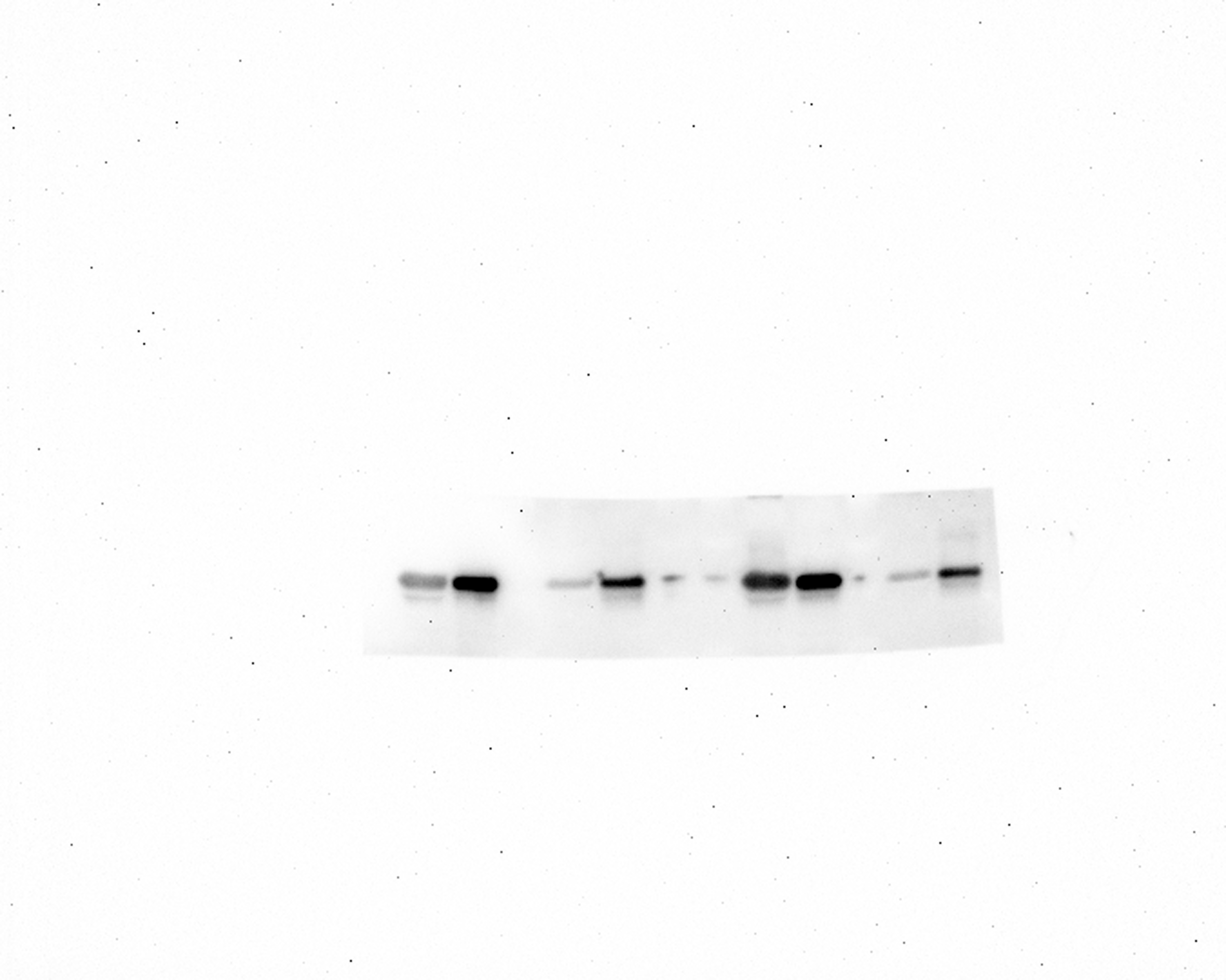

Supplement: Source data 1. [file elife-69734-data1.zip › Vasileva_Source Data/Figure 4-source data 1]

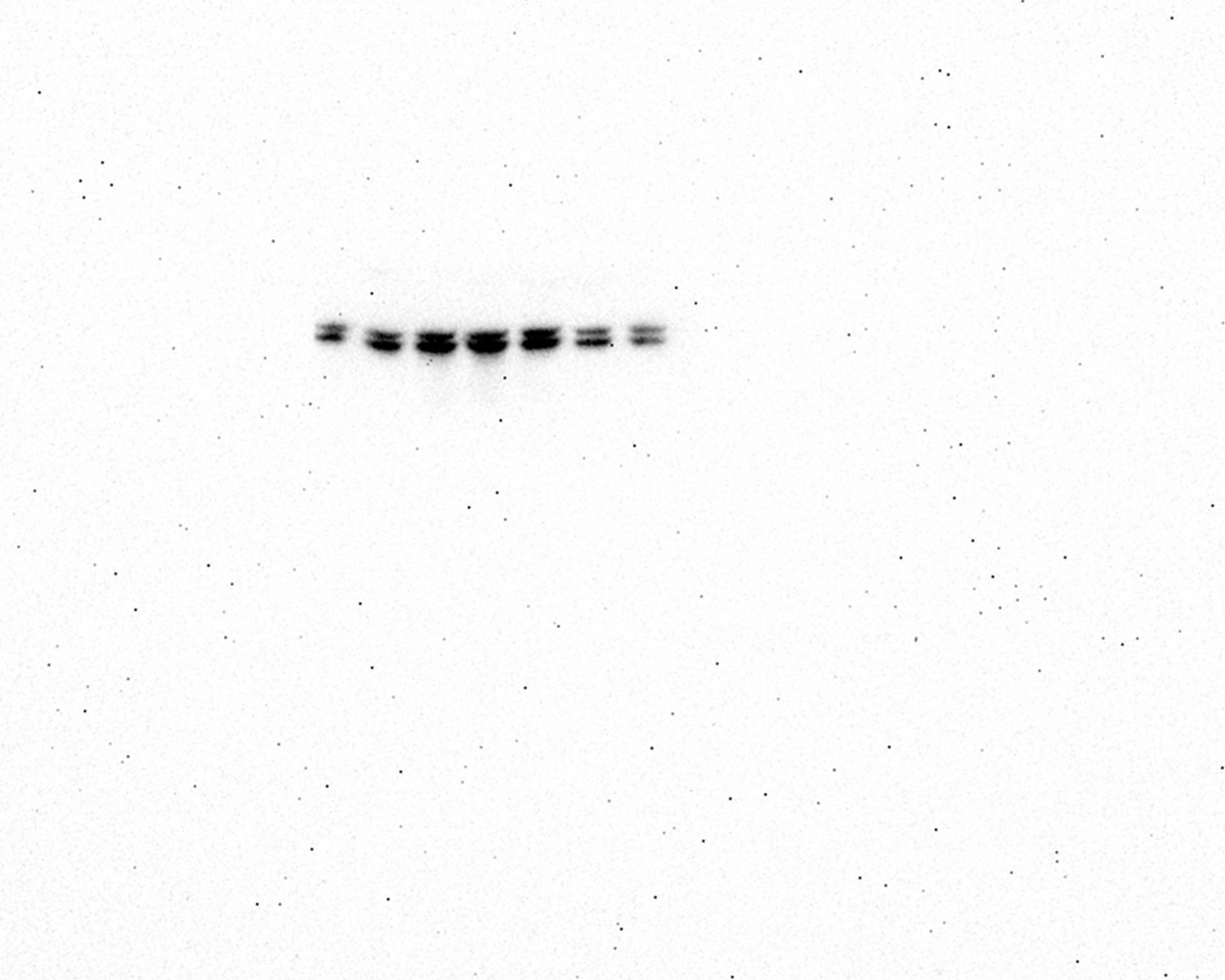

Supplement: Source data 1. [file elife-69734-data1.zip › Vasileva_Source Data/Figure 6-source data 1.jpg]

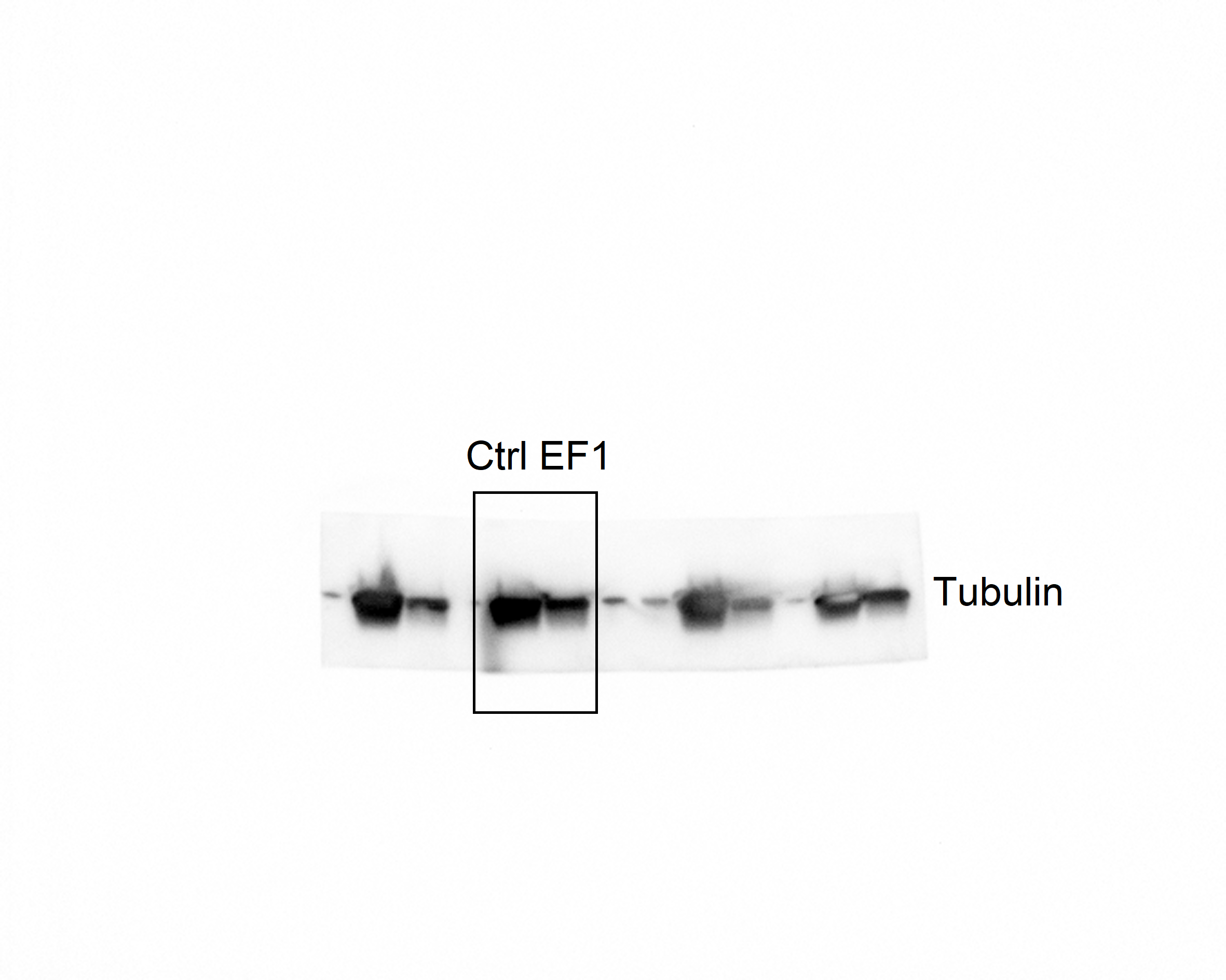

Supplement: Source data 1. [file elife-69734-data1.zip › Vasileva_Source Data/Figure 4-source data 6.tif]

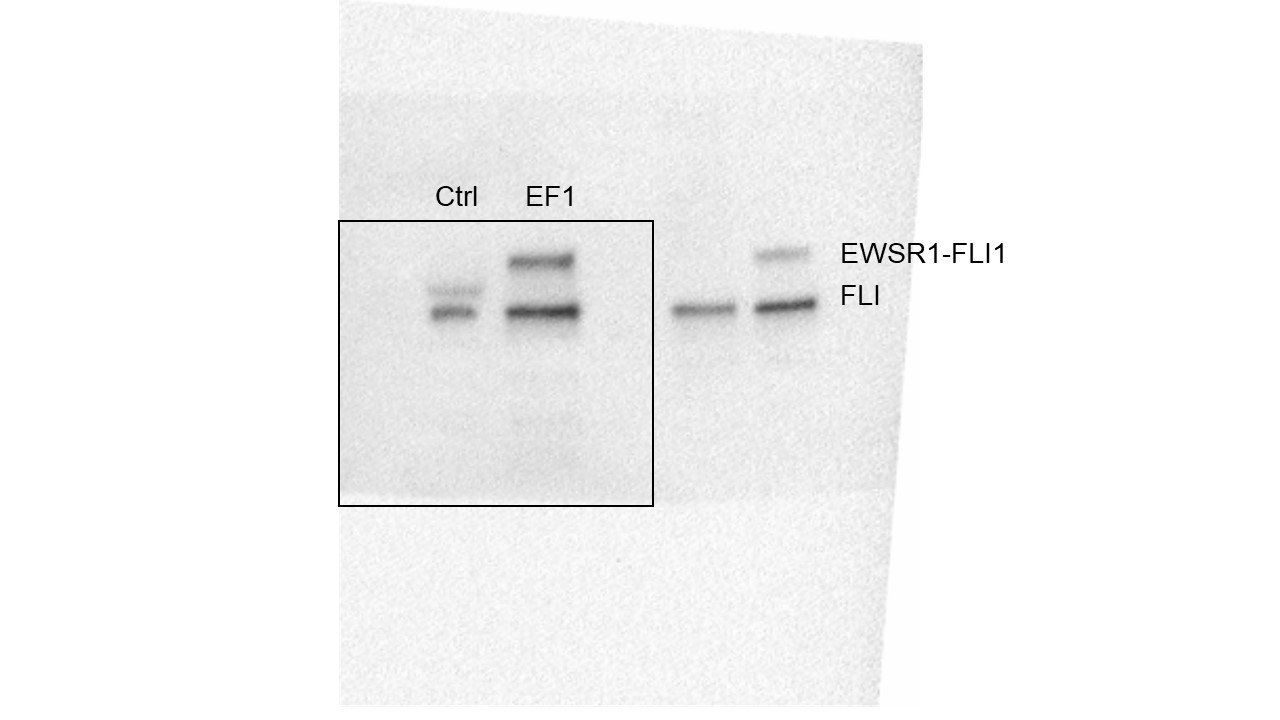

Supplement: Source data 1. [file elife-69734-data1.zip › Vasileva_Source Data/Figure 2-source data 4.jpg]

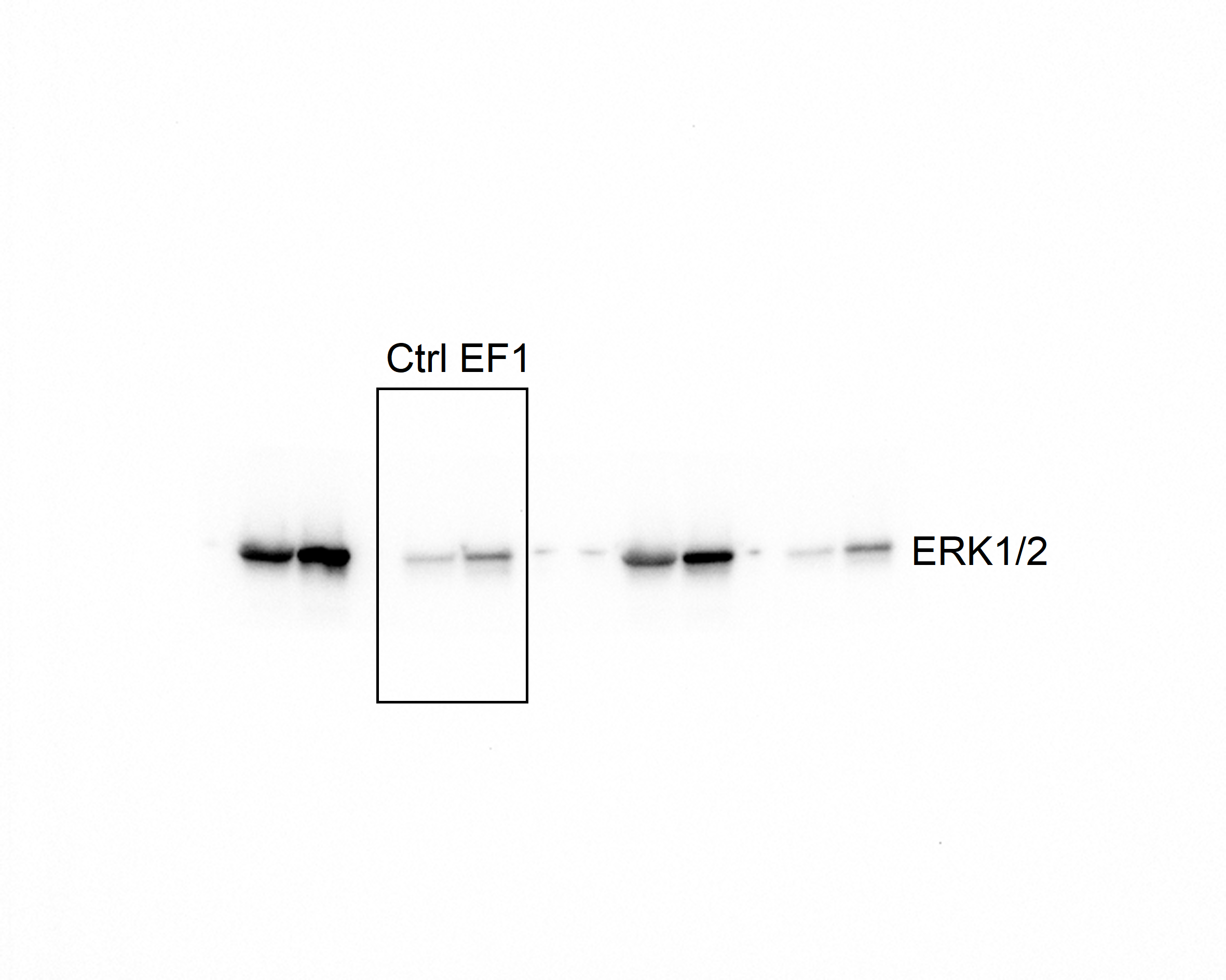

Supplement: Source data 1. [file elife-69734-data1.zip › Vasileva_Source Data/Figure 4-source data 4.tif]

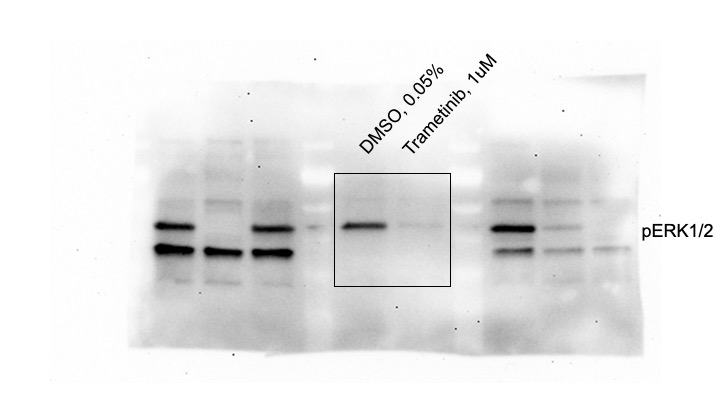

Supplement: Source data 1. [file elife-69734-data1.zip › Vasileva_Source Data/Figure 7-figure supplement 1-source data 6.jpeg]

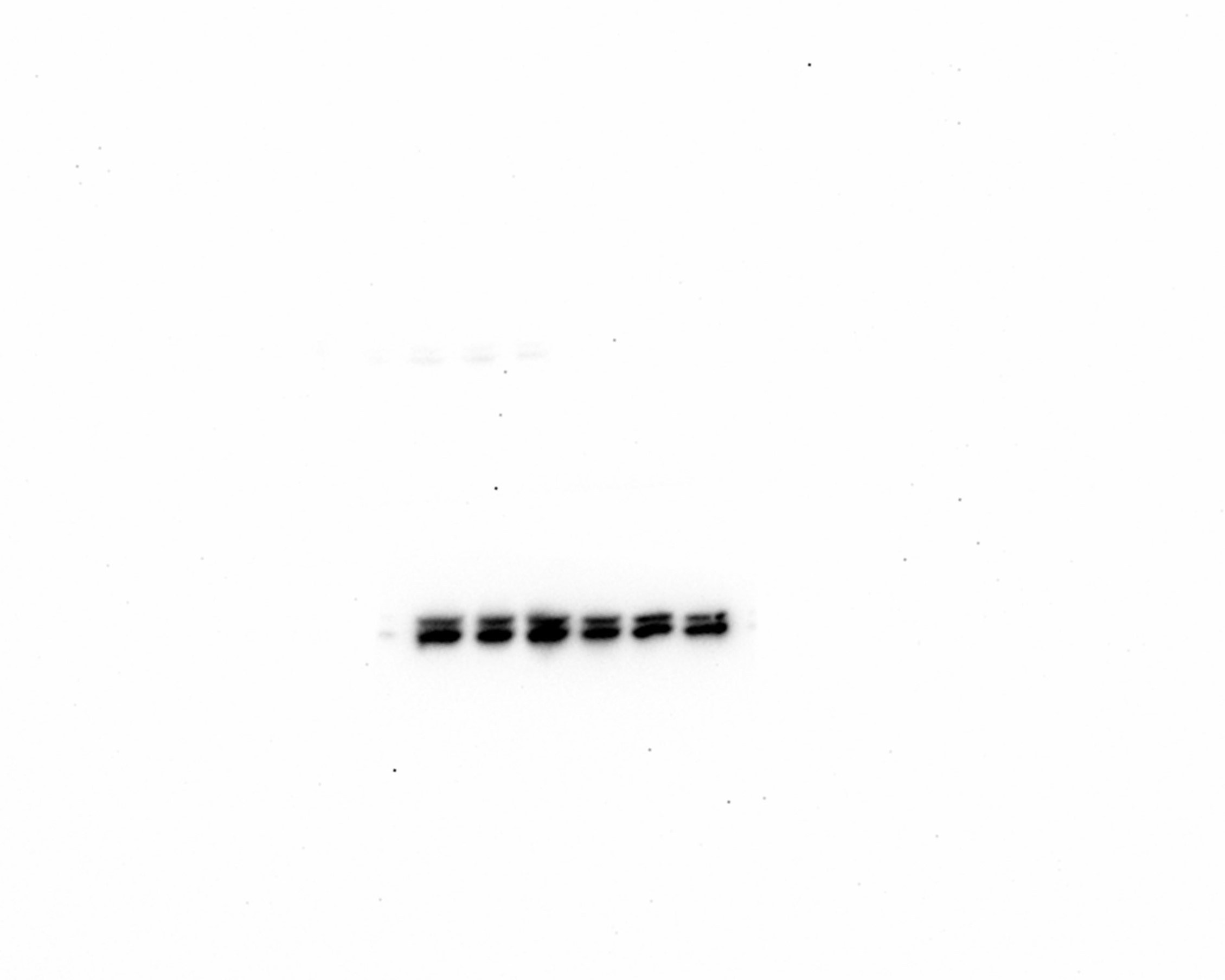

Supplement: Source data 1. [file elife-69734-data1.zip › Vasileva_Source Data/Figure 6-source data 3.jpg]

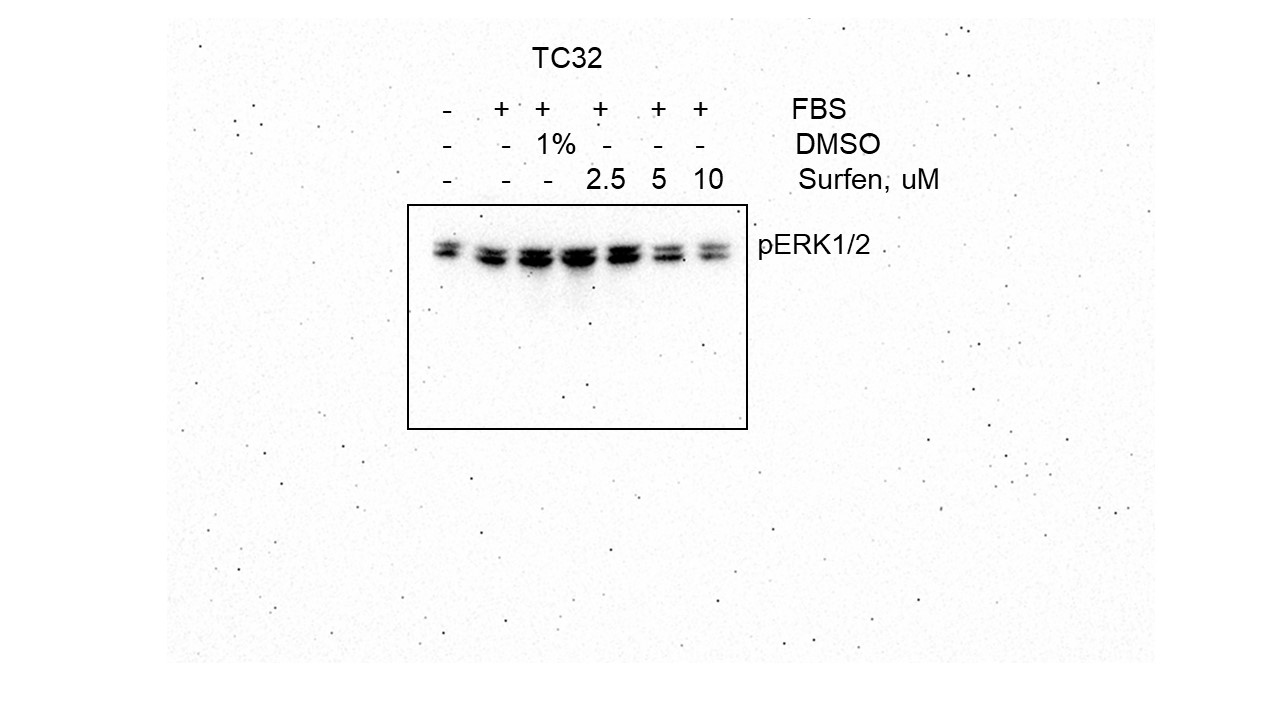

Supplement: Source data 1. [file elife-69734-data1.zip › Vasileva_Source Data/Figure 6-source data 2.jpg]

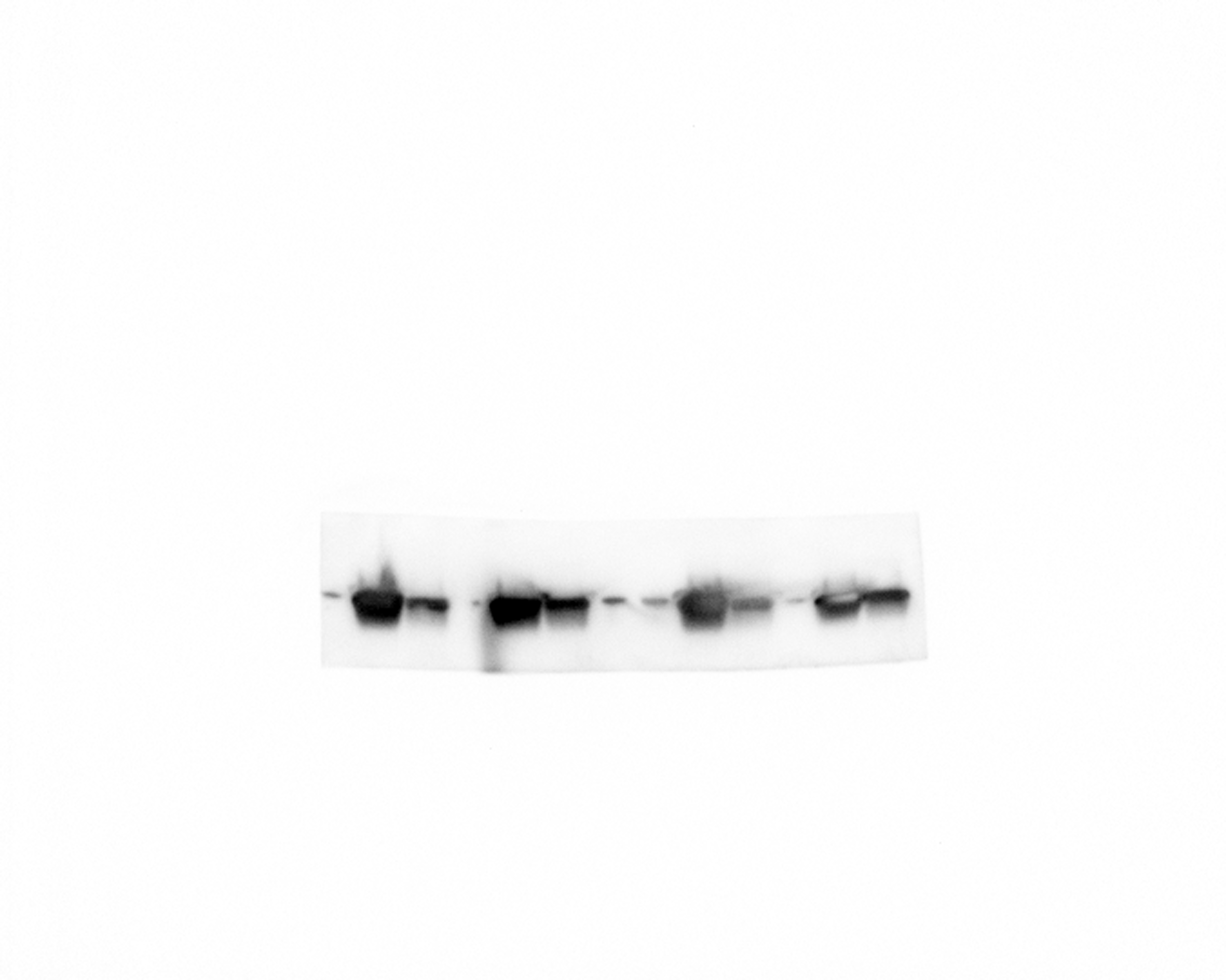

Supplement: Source data 1. [file elife-69734-data1.zip › Vasileva_Source Data/Figure 4-source data 5.tif]

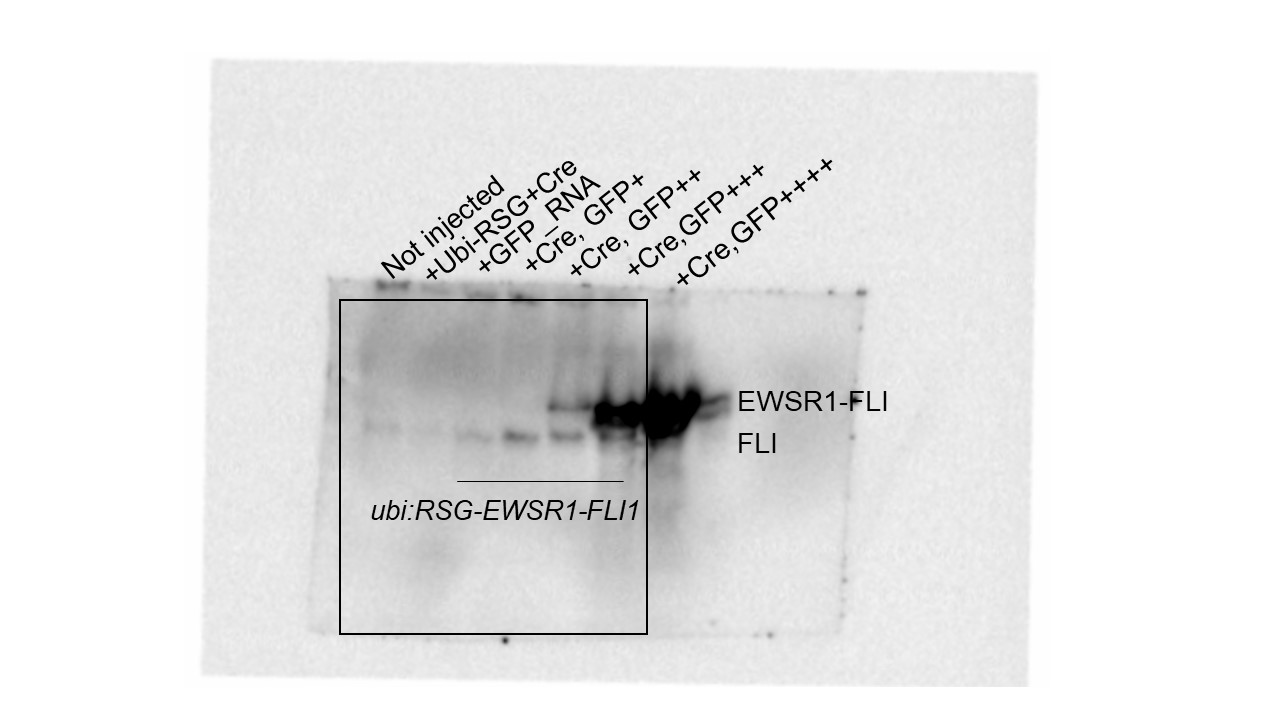

Supplement: Source data 1. [file elife-69734-data1.zip › Vasileva_Source Data/Figure 3-source data 1.jpg]

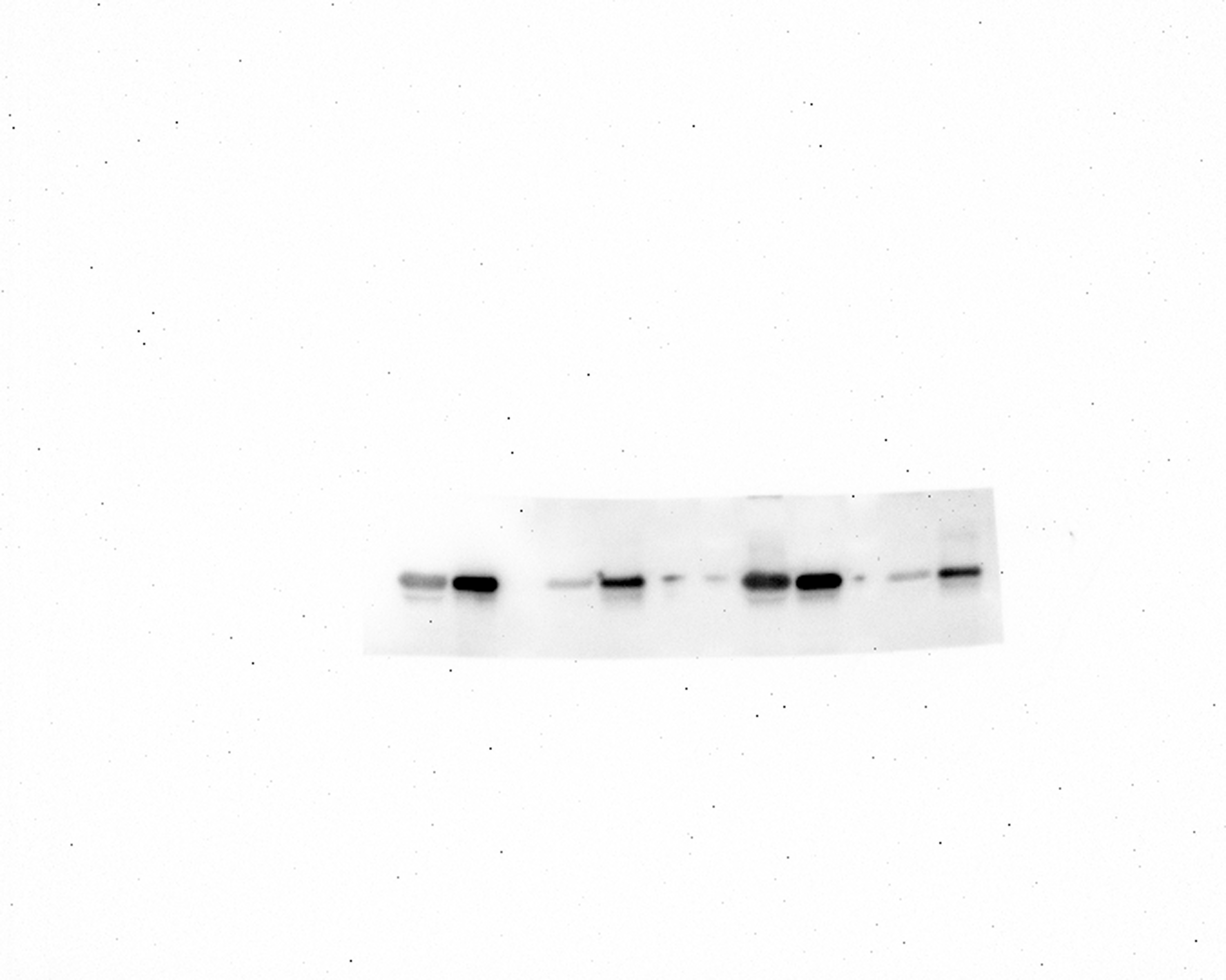

Supplement: Source data 1. [file elife-69734-data1.zip › Vasileva_Source Data/Figure 4-source data 1.tif]

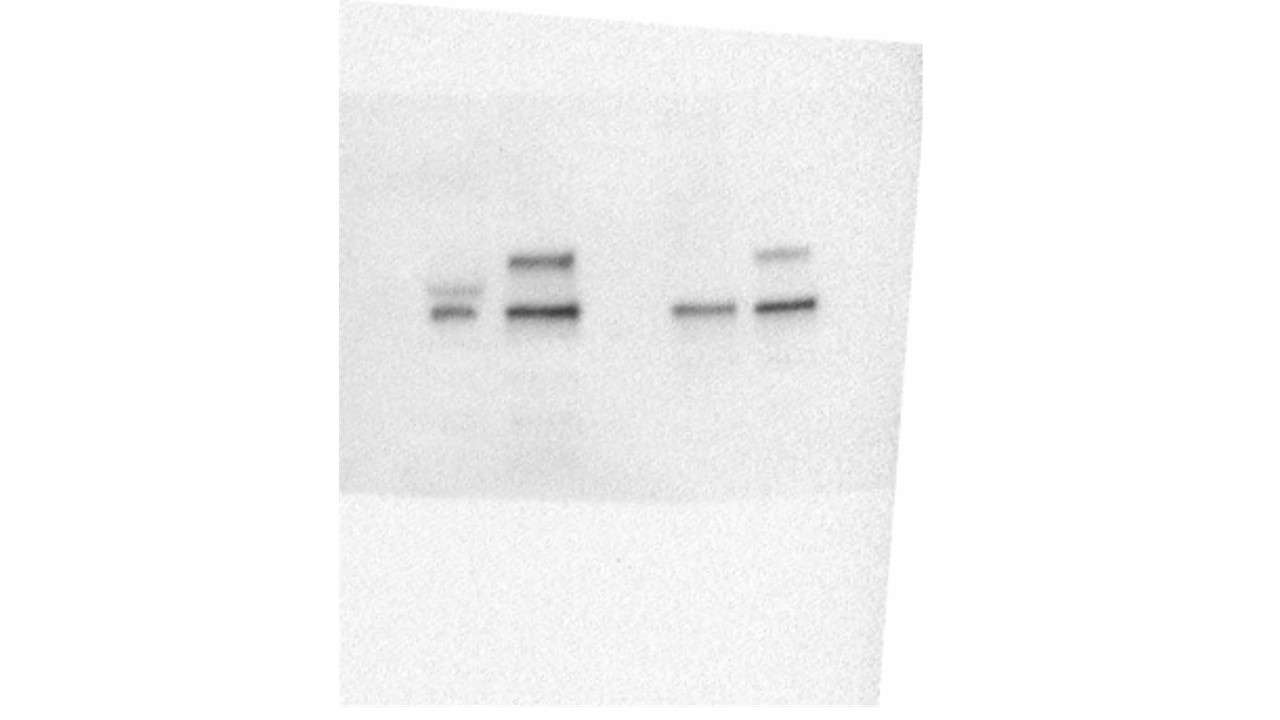

Supplement: Source data 1. [file elife-69734-data1.zip › Vasileva_Source Data/Figure 2-source data 3.jpg]

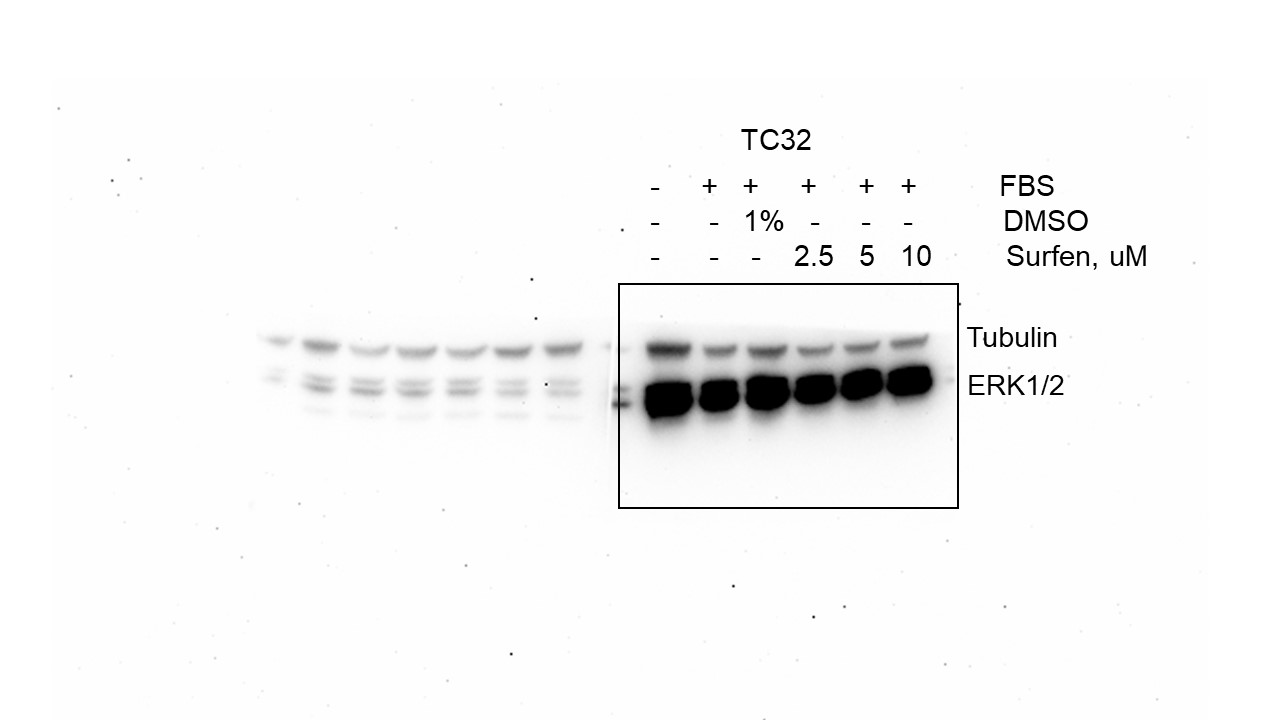

Supplement: Source data 1. [file elife-69734-data1.zip › Vasileva_Source Data/Figure 6-source data 6.jpg]

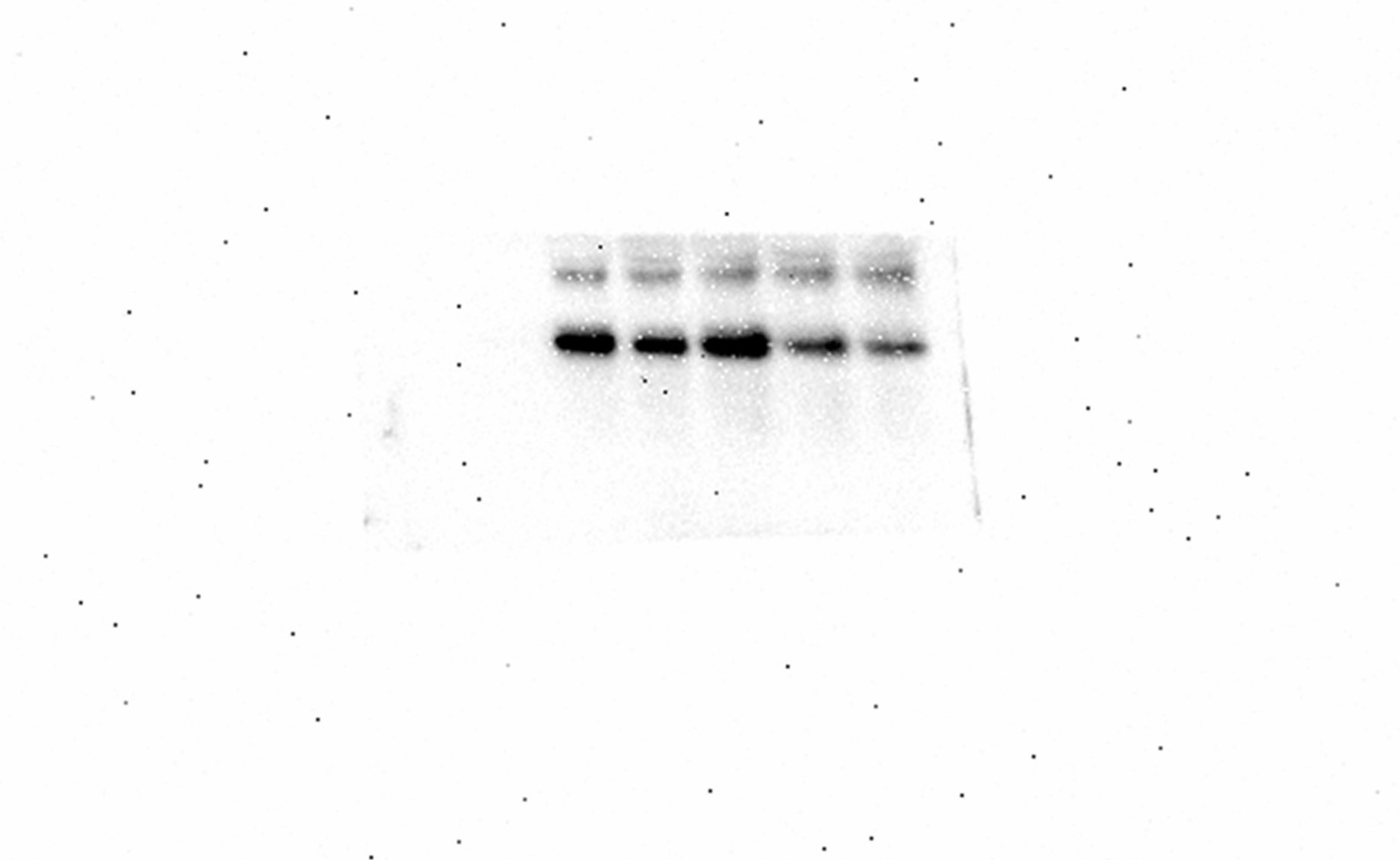

Supplement: Source data 1. [file elife-69734-data1.zip › Vasileva_Source Data/Figure 6-source data 7.jpg]

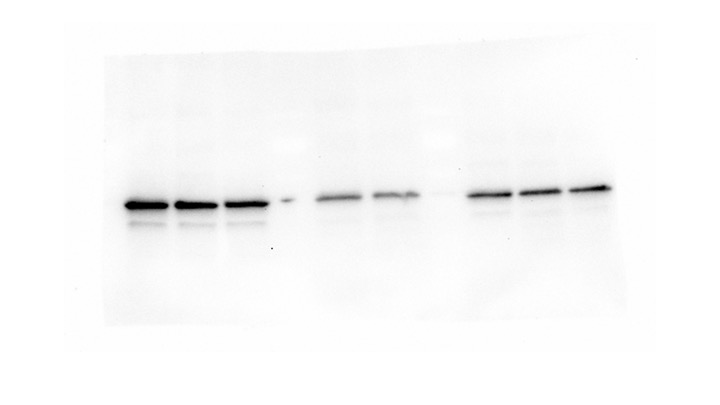

Supplement: Source data 1. [file elife-69734-data1.zip › Vasileva_Source Data/Figure 7-figure supplement 1-source data 7.jpeg]

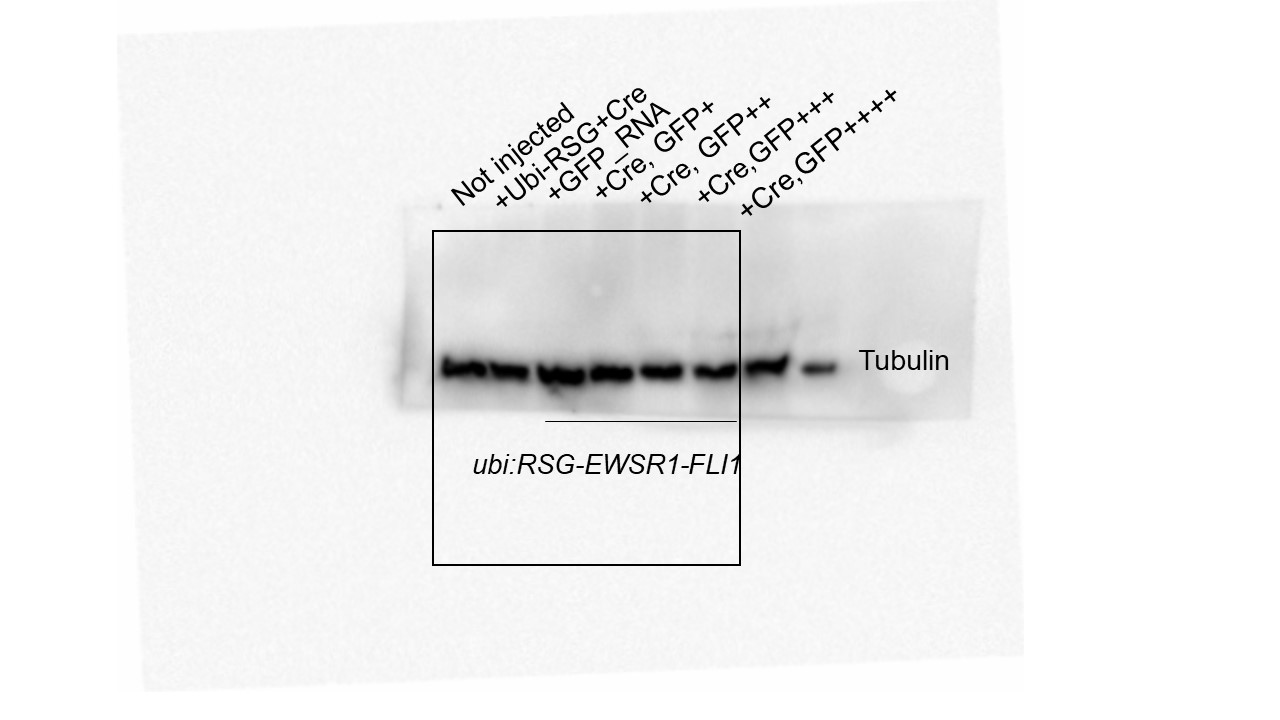

Supplement: Source data 1. [file elife-69734-data1.zip › Vasileva_Source Data/Figure 3-source data 4.jpg]

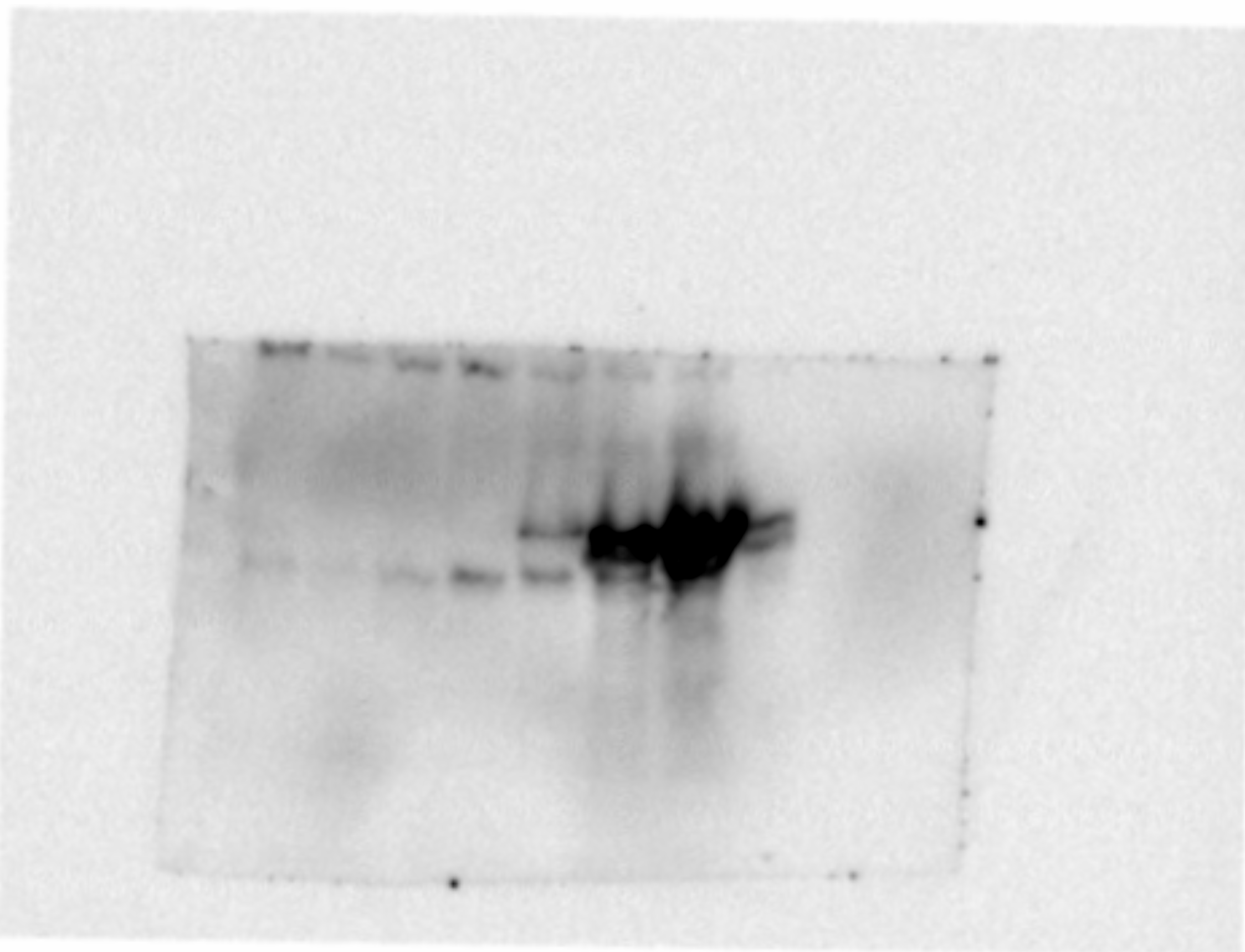

Supplement: Source data 1. [file elife-69734-data1.zip › Vasileva_Source Data/Figure 3-source data 1]

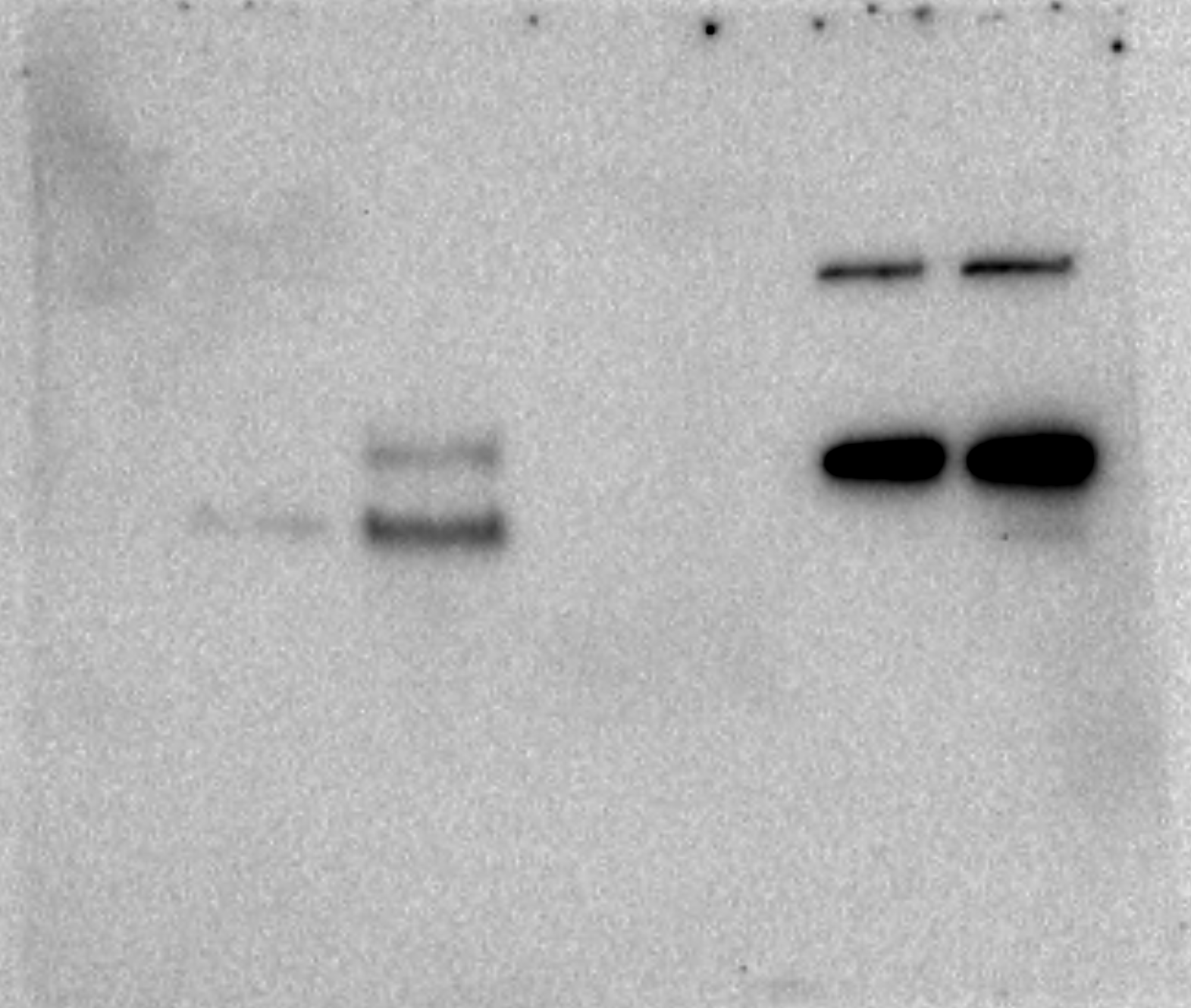

Supplement: Source data 1. [file elife-69734-data1.zip › Vasileva_Source Data/Figure 1-figure supplement 2-source data 1]

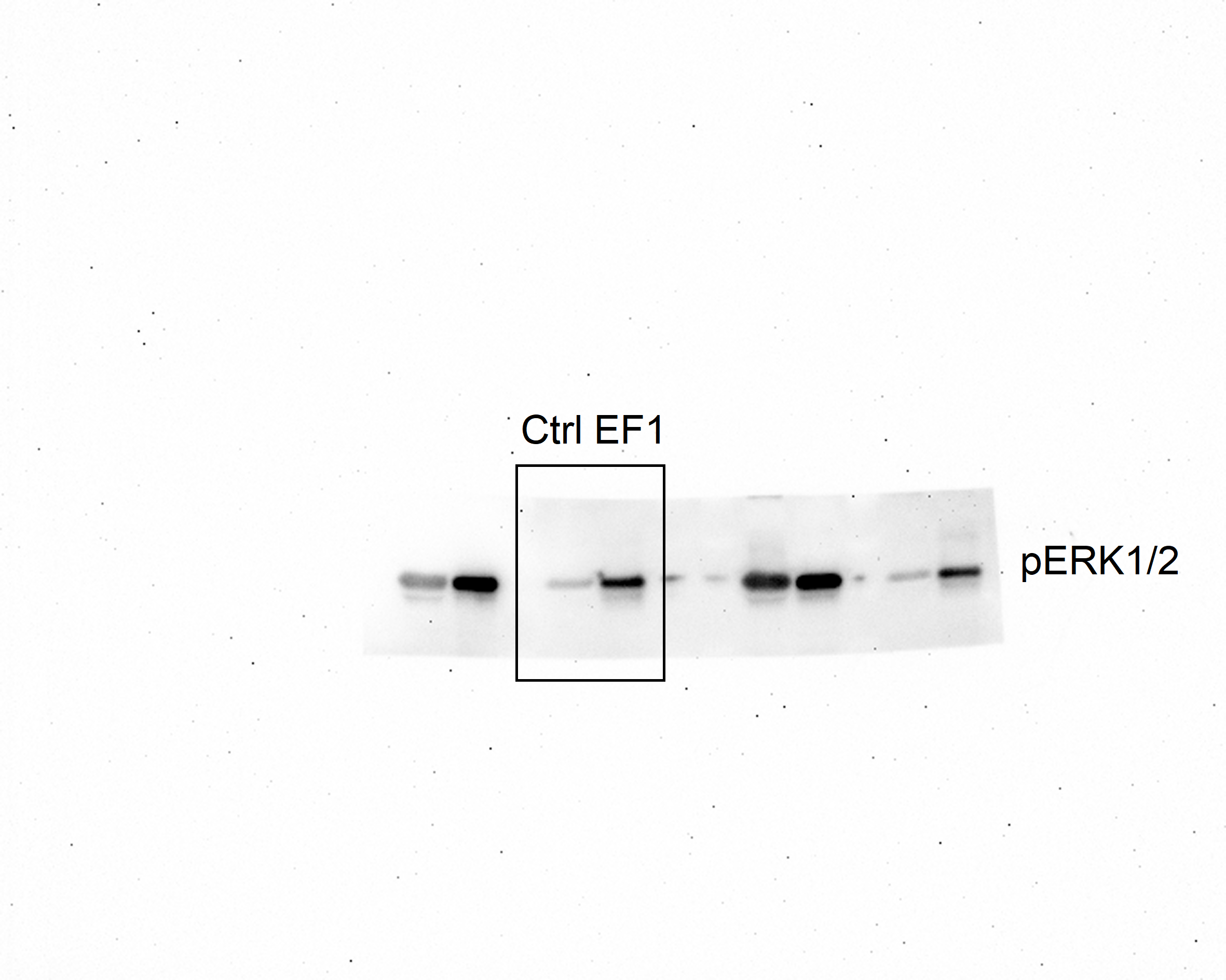

Supplement: Source data 1. [file elife-69734-data1.zip › Vasileva_Source Data/Figure 4-source data 2.tif]

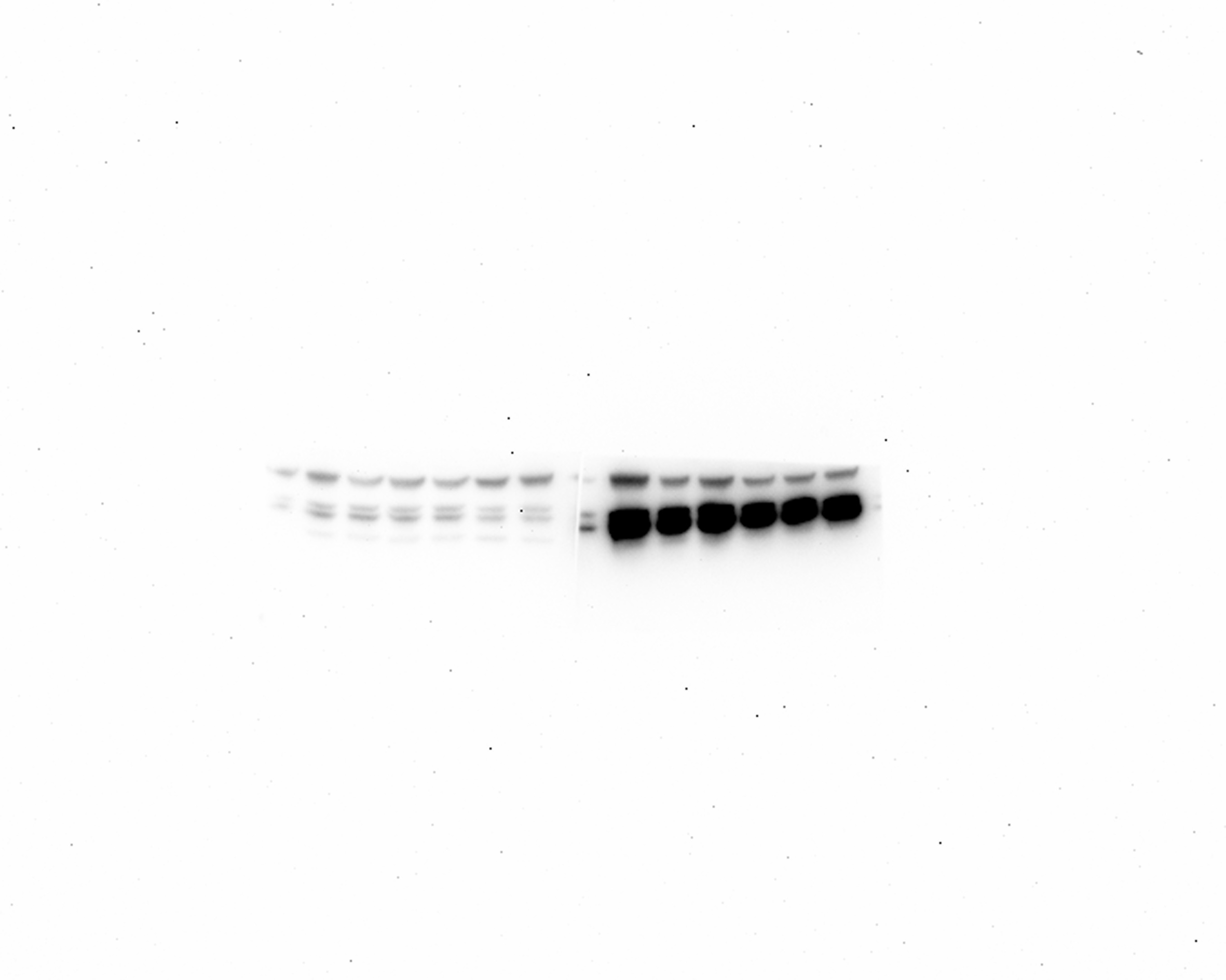

Supplement: Source data 1. [file elife-69734-data1.zip › Vasileva_Source Data/Figure 6-source data 5.jpg]

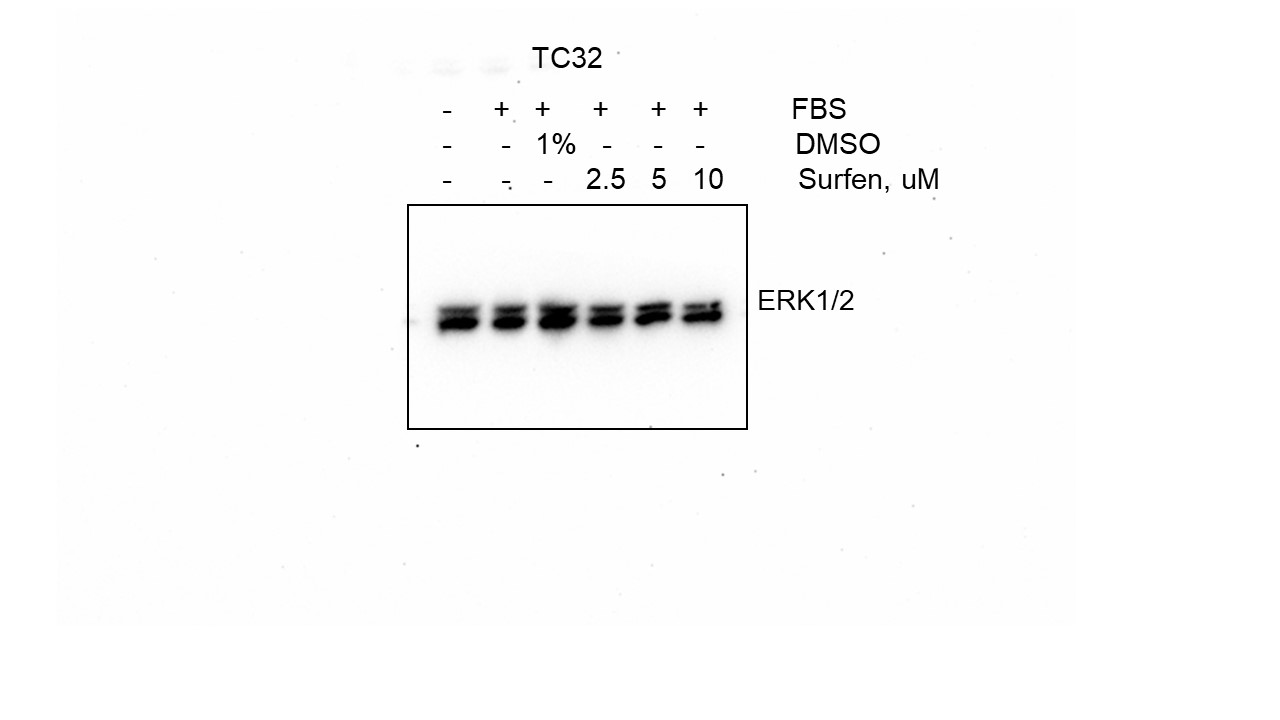

Supplement: Source data 1. [file elife-69734-data1.zip › Vasileva_Source Data/Figure 6-source data 4.jpg]

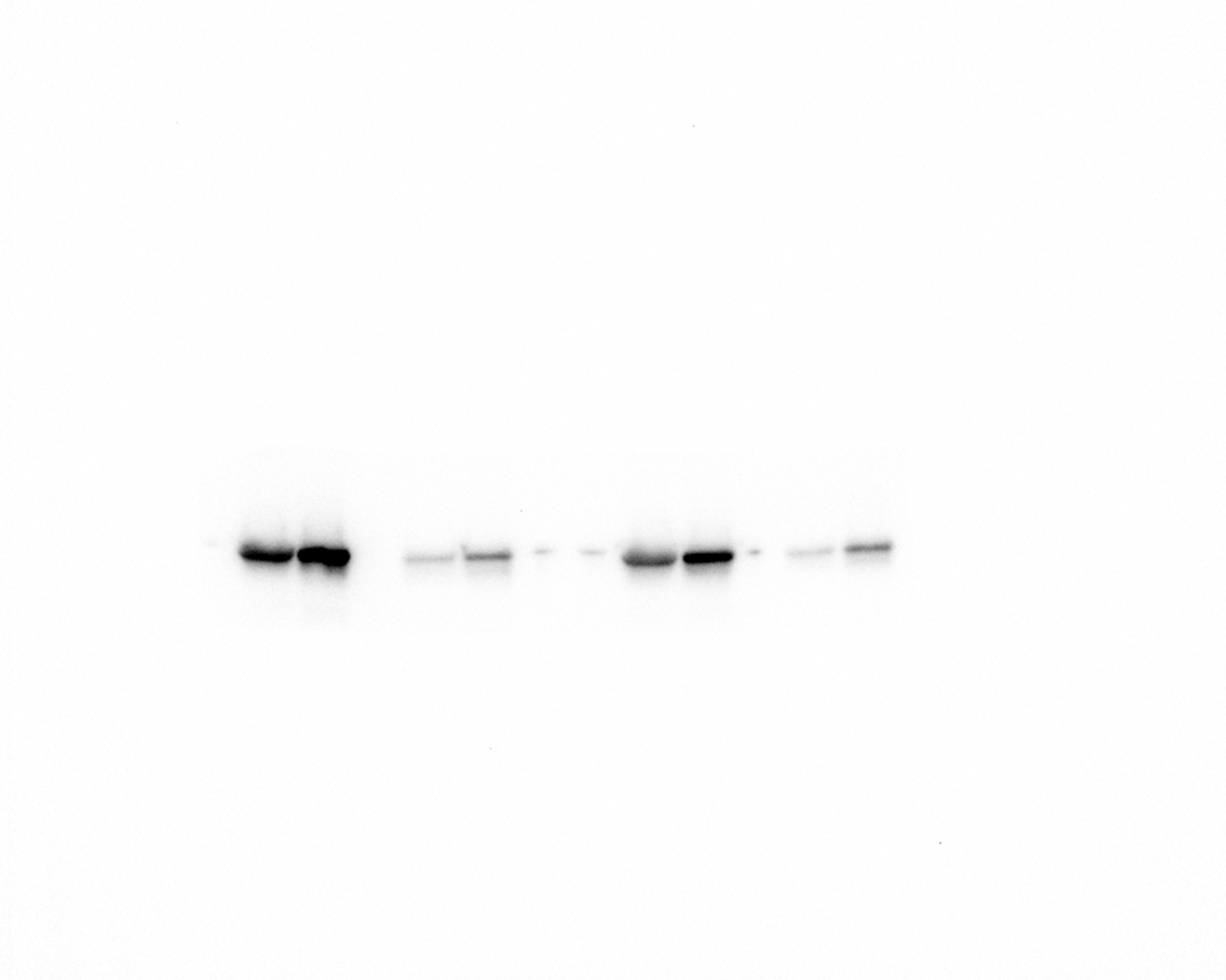

Supplement: Source data 1. [file elife-69734-data1.zip › Vasileva_Source Data/Figure 4-source data 3.tif]

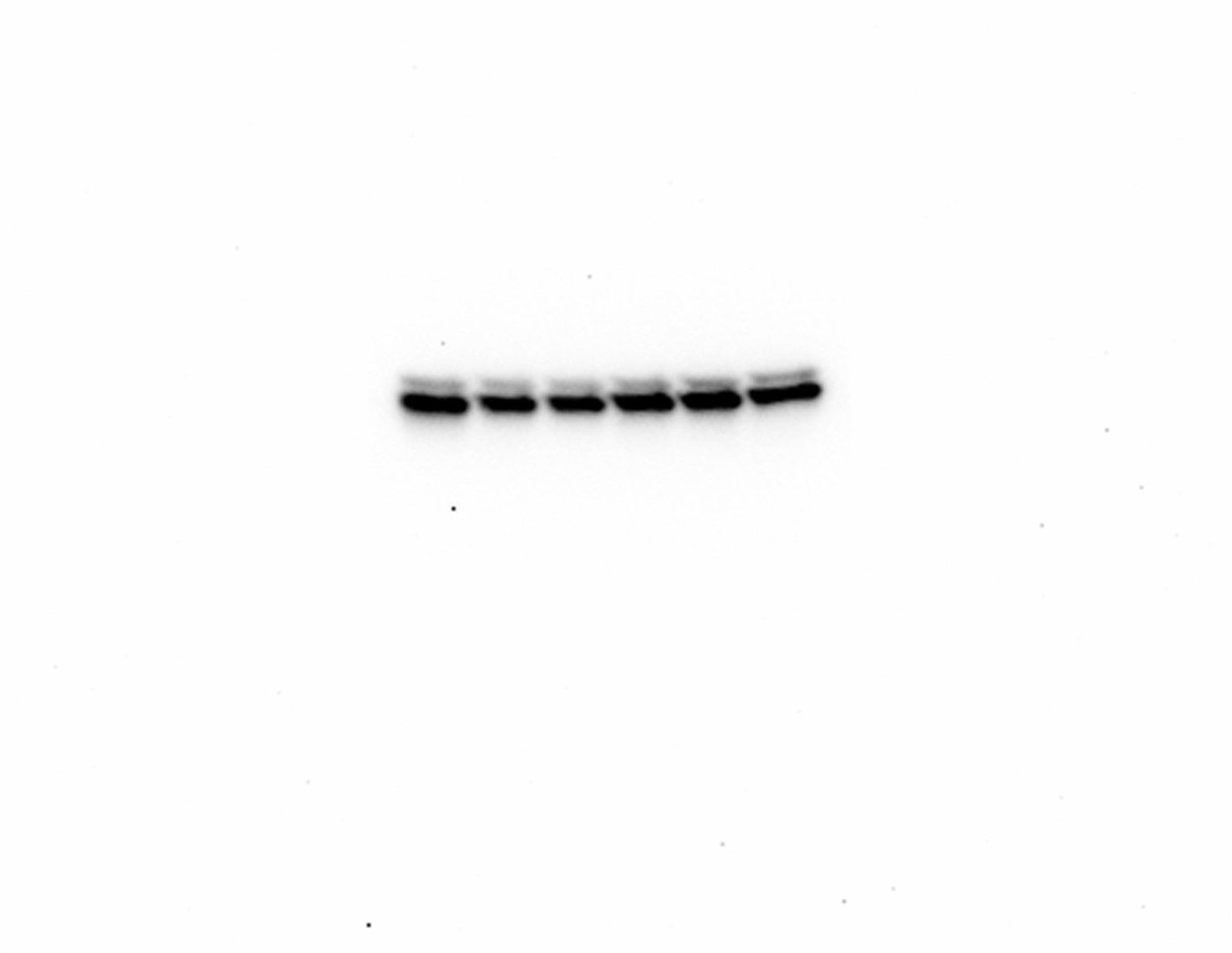

Supplement: Source data 1. [file elife-69734-data1.zip › Vasileva_Source Data/Figure 6-source data 9.jpg]

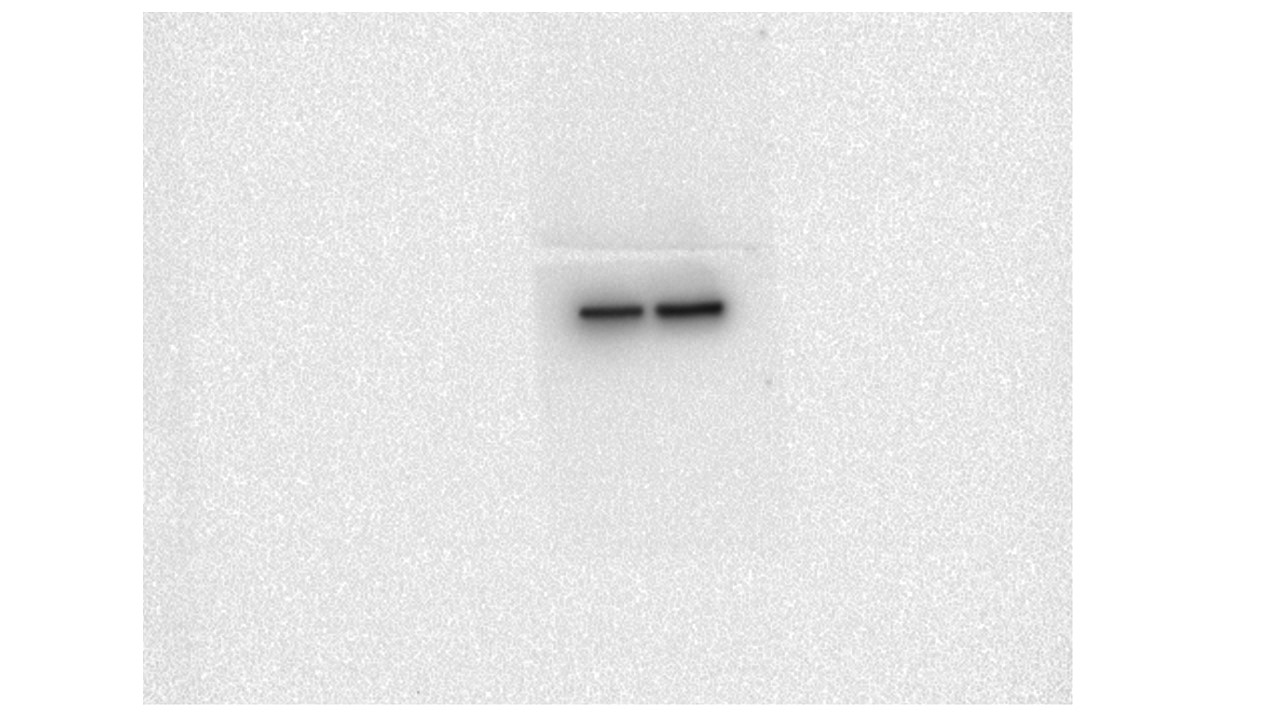

Supplement: Source data 1. [file elife-69734-data1.zip › Vasileva_Source Data/Figure 1-figure supplement 2-source data 3.jpg]

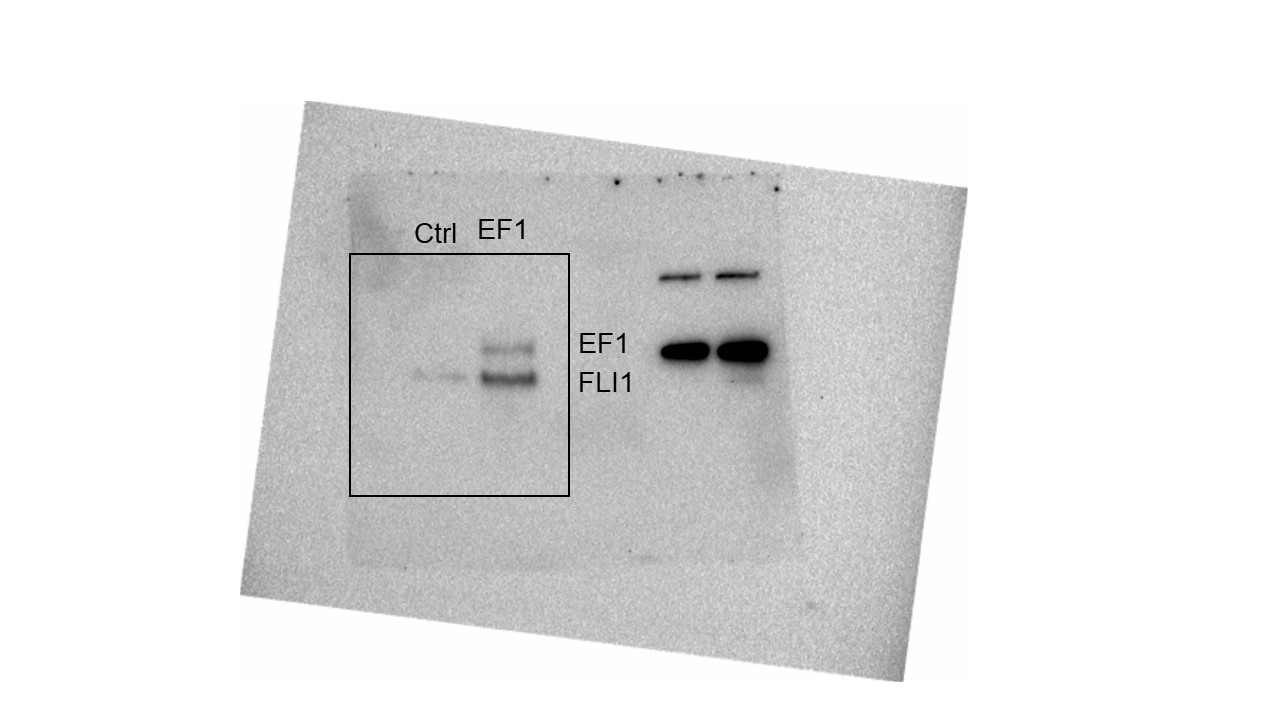

Supplement: Source data 1. [file elife-69734-data1.zip › Vasileva_Source Data/Figure 1-figure supplement 2-source data 2.jpg]

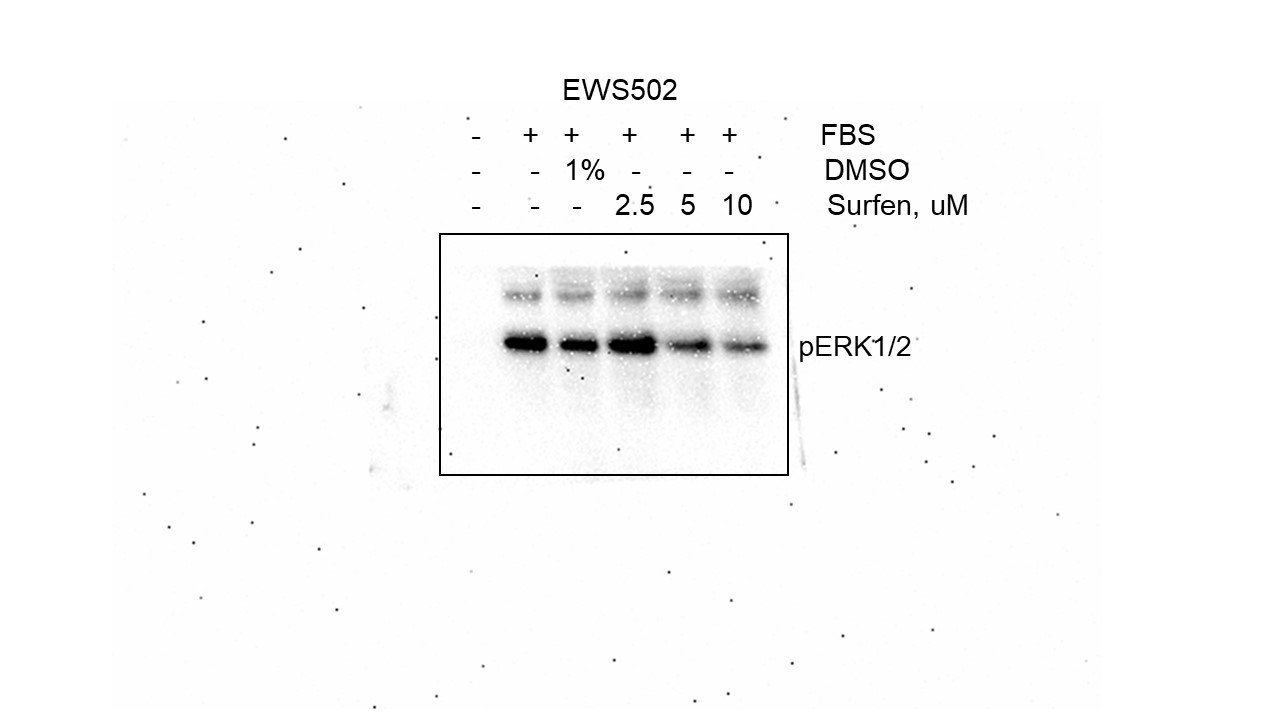

Supplement: Source data 1. [file elife-69734-data1.zip › Vasileva_Source Data/Figure 6-source data 8.jpg]

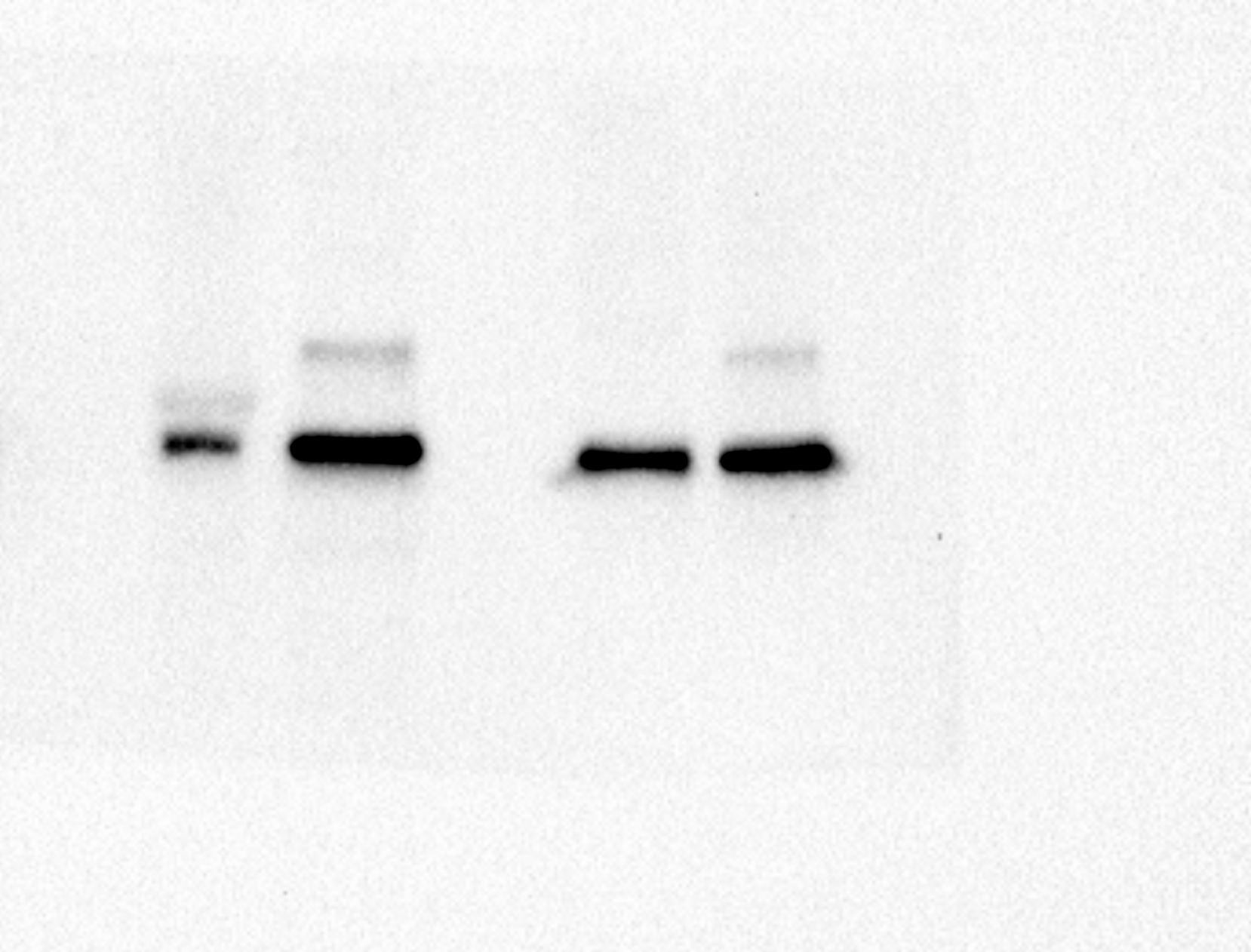

Supplement: Source data 1. [file elife-69734-data1.zip › Vasileva_Source Data/Figure 2-source data 7]

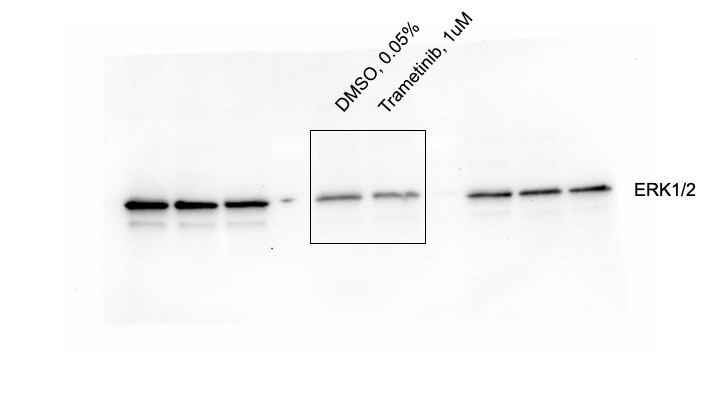

Supplement: Source data 1. [file elife-69734-data1.zip › Vasileva_Source Data/Figure 7-figure supplement 1-source data 8.jpeg]

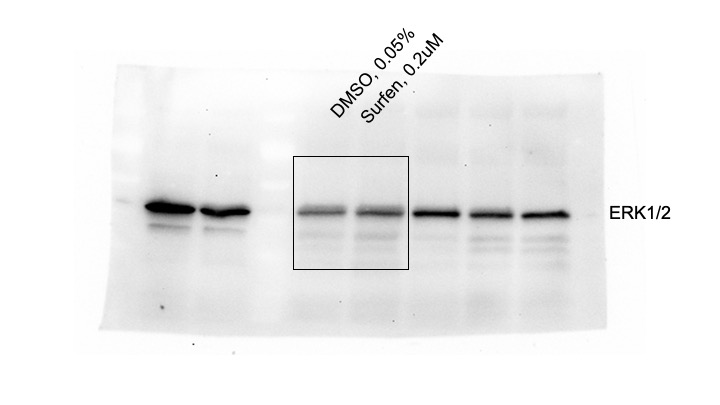

Supplement: Source data 1. [file elife-69734-data1.zip › Vasileva_Source Data/Figure 7-figure supplement 1-source data 4.jpeg]

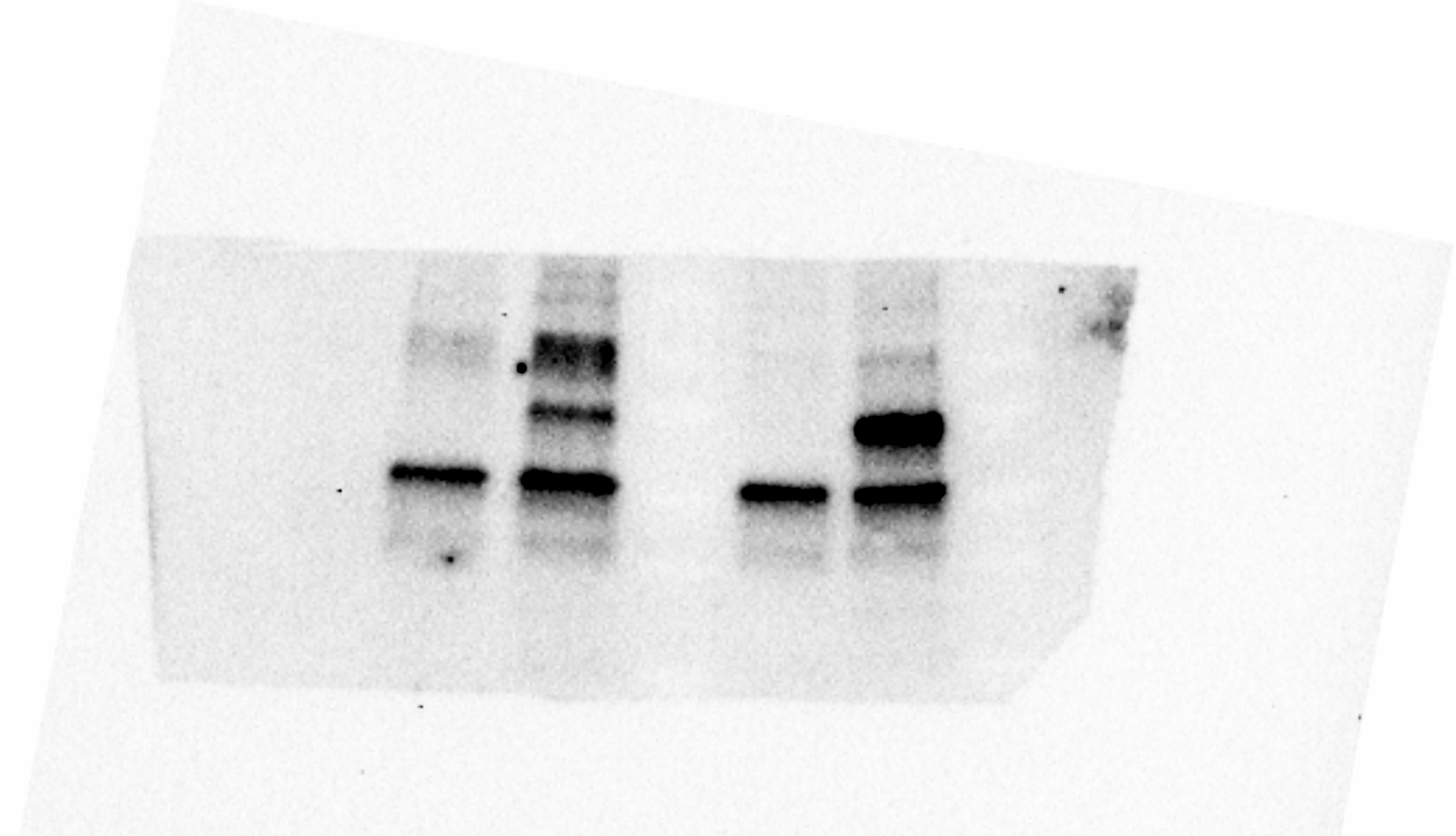

Supplement: Source data 1. [file elife-69734-data1.zip › Vasileva_Source Data/Figure 2-source data 1]

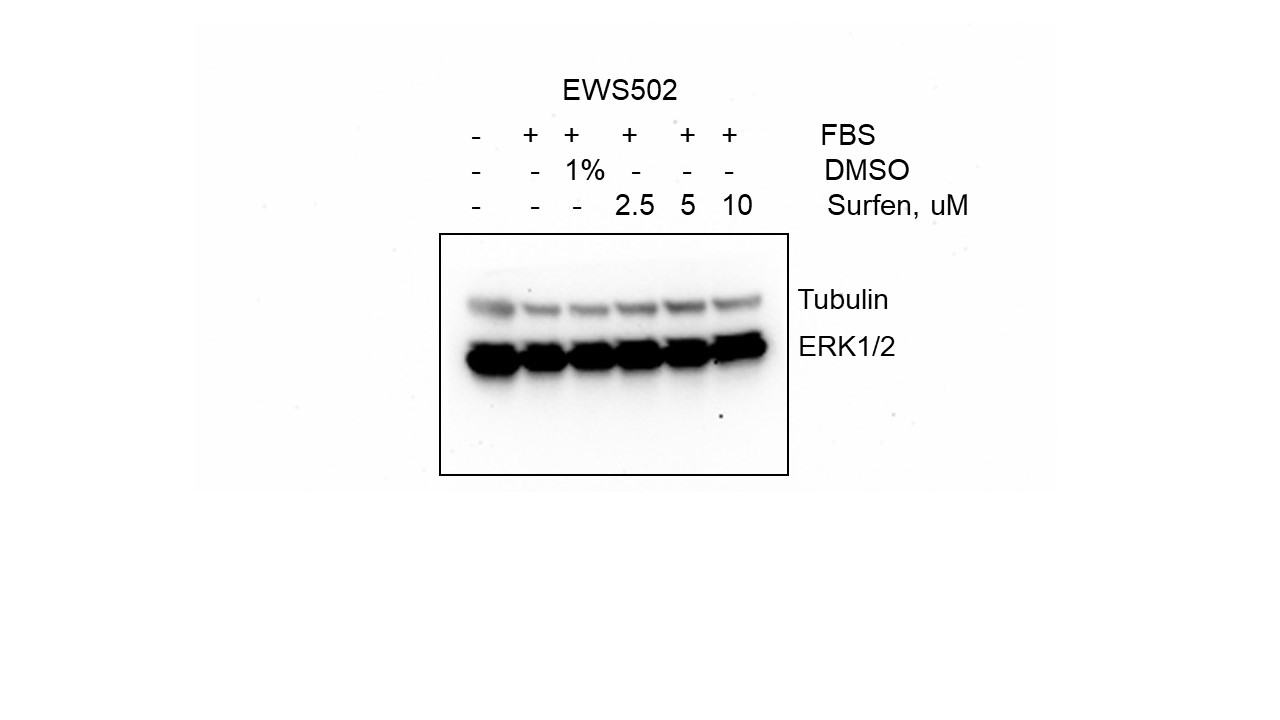

Supplement: Source data 1. [file elife-69734-data1.zip › Vasileva_Source Data/Figure 6-source data 12.jpg]

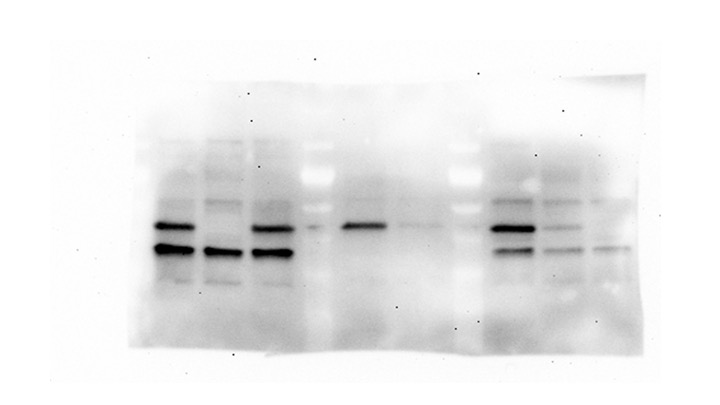

Supplement: Source data 1. [file elife-69734-data1.zip › Vasileva_Source Data/Figure 7-figure supplement 1-source data 5.jpeg]

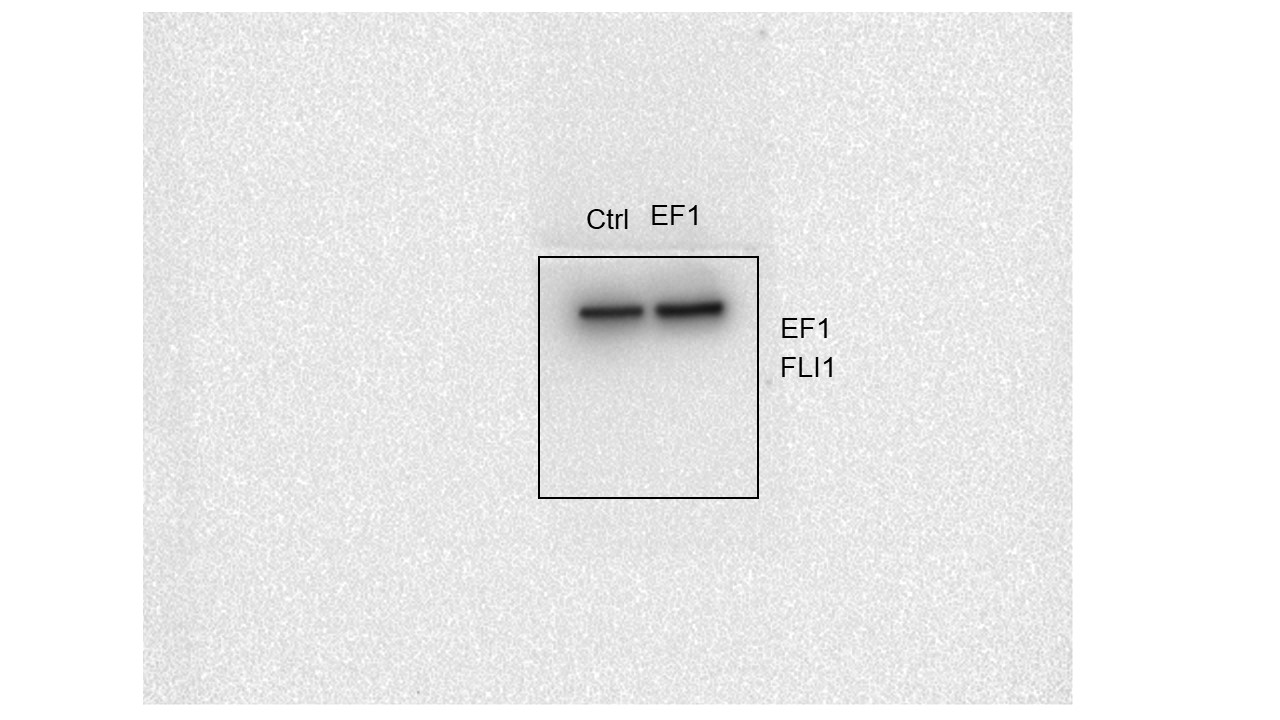

Supplement: Source data 1. [file elife-69734-data1.zip › Vasileva_Source Data/Figure 1-figure supplement 2-source data 4.jpg]

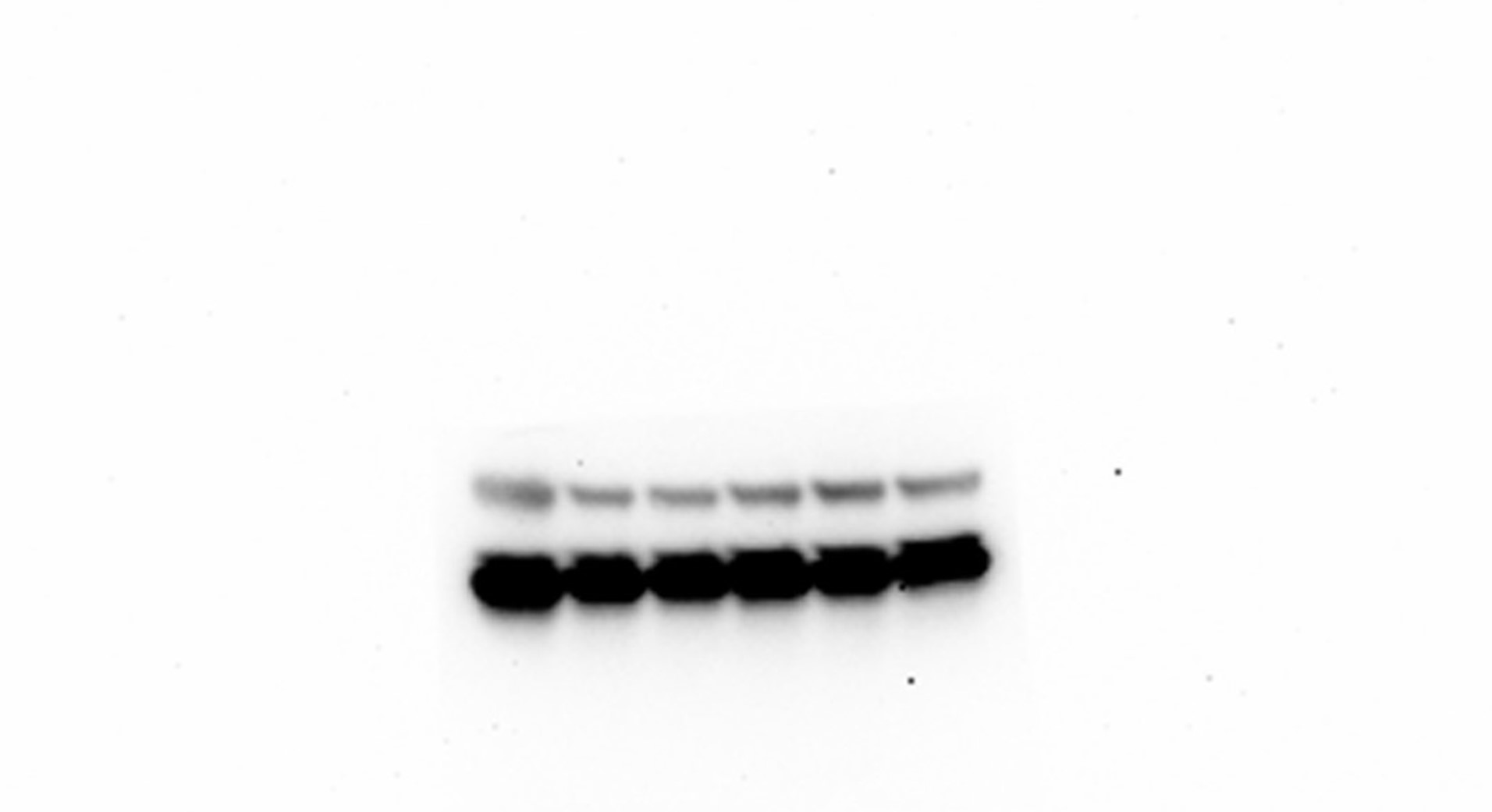

Supplement: Source data 1. [file elife-69734-data1.zip › Vasileva_Source Data/Figure 6-source data 11.jpg]

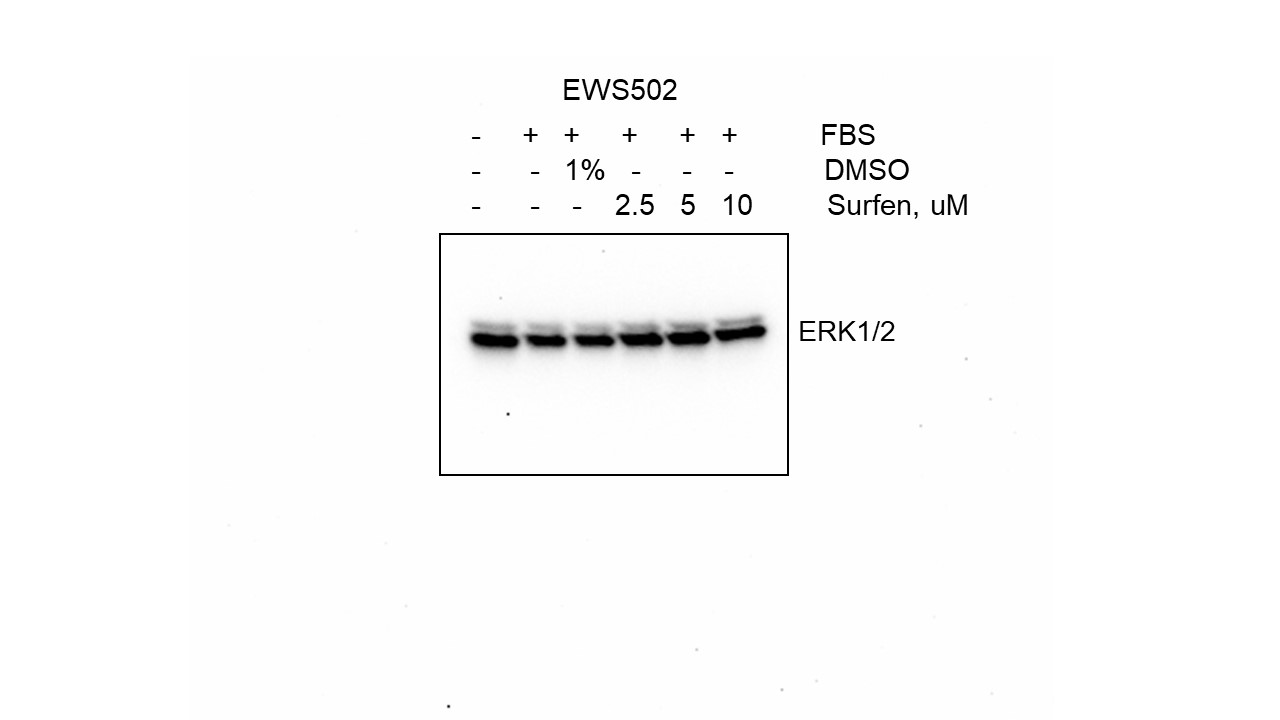

Supplement: Source data 1. [file elife-69734-data1.zip › Vasileva_Source Data/Figure 6-source data 10.jpg]

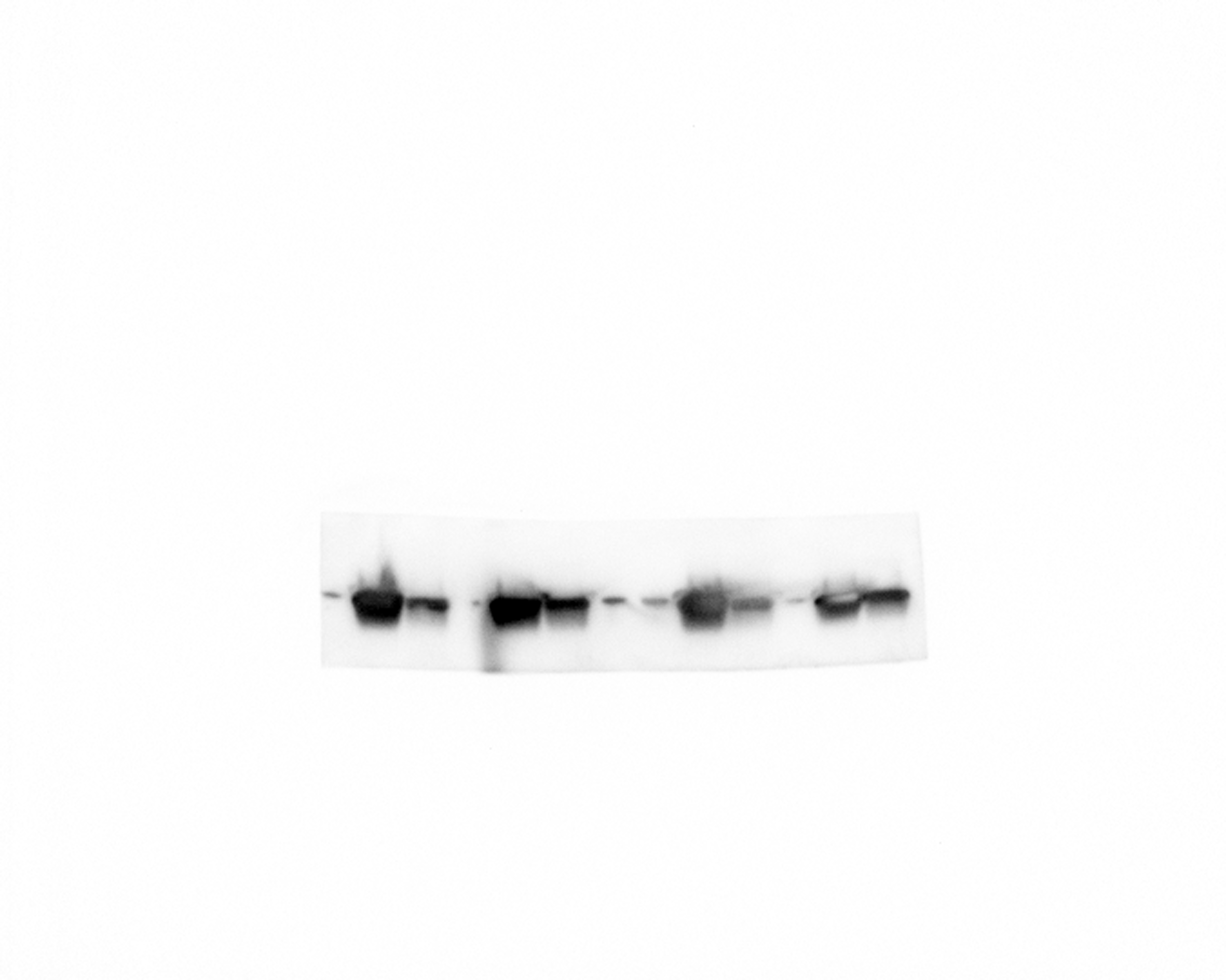

Supplement: Source data 1. [file elife-69734-data1.zip › Vasileva_Source Data/Figure 4-source data 5]

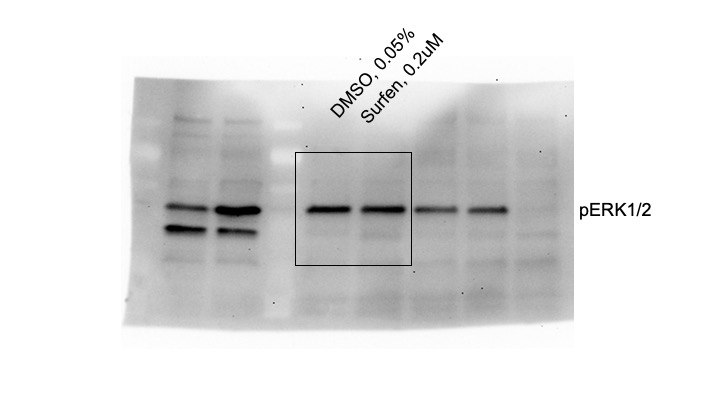

Supplement: Source data 1. [file elife-69734-data1.zip › Vasileva_Source Data/Figure 7-figure supplement 1-source data 2.jpeg]

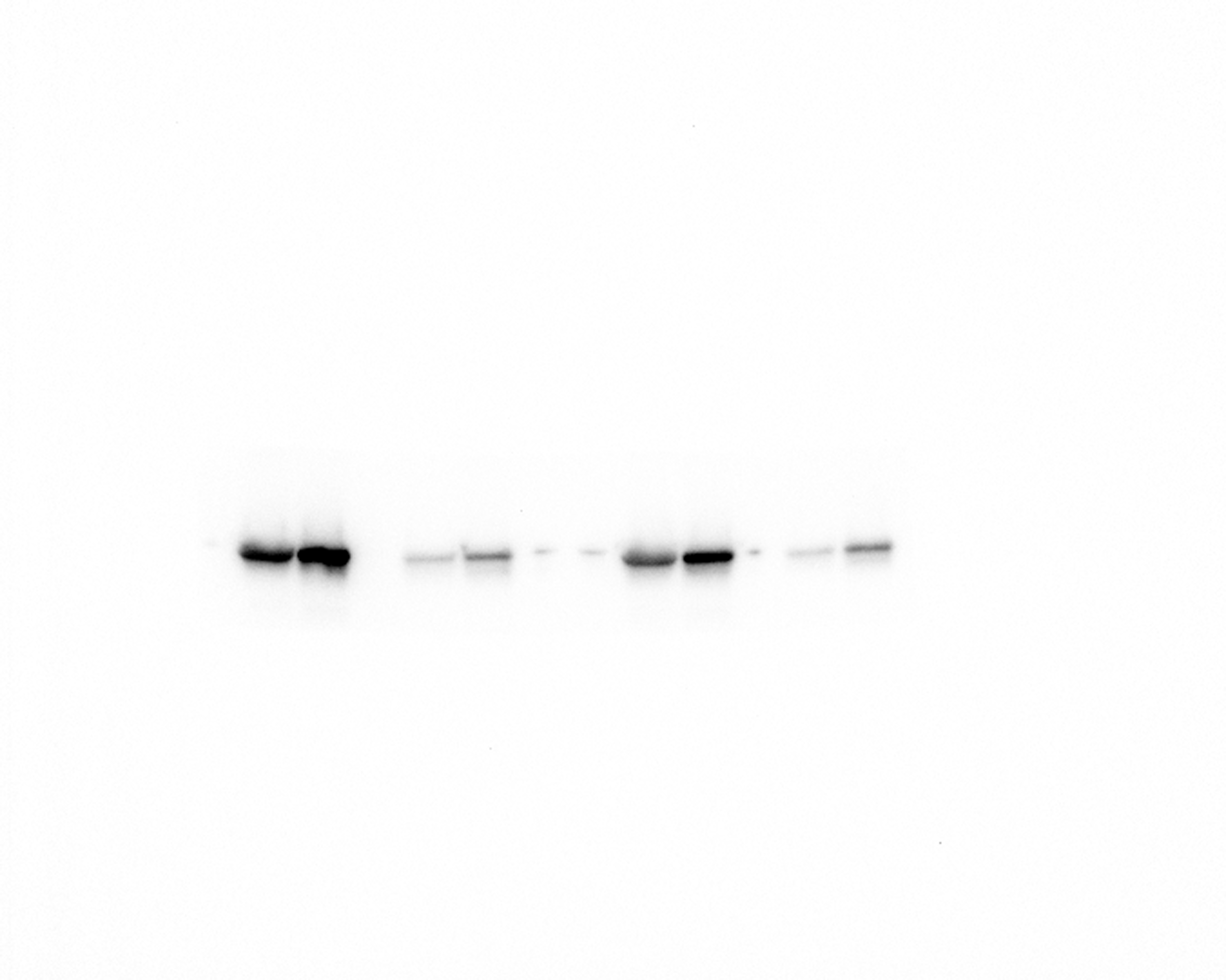

Supplement: Source data 1. [file elife-69734-data1.zip › Vasileva_Source Data/Figure 4-source data 3]

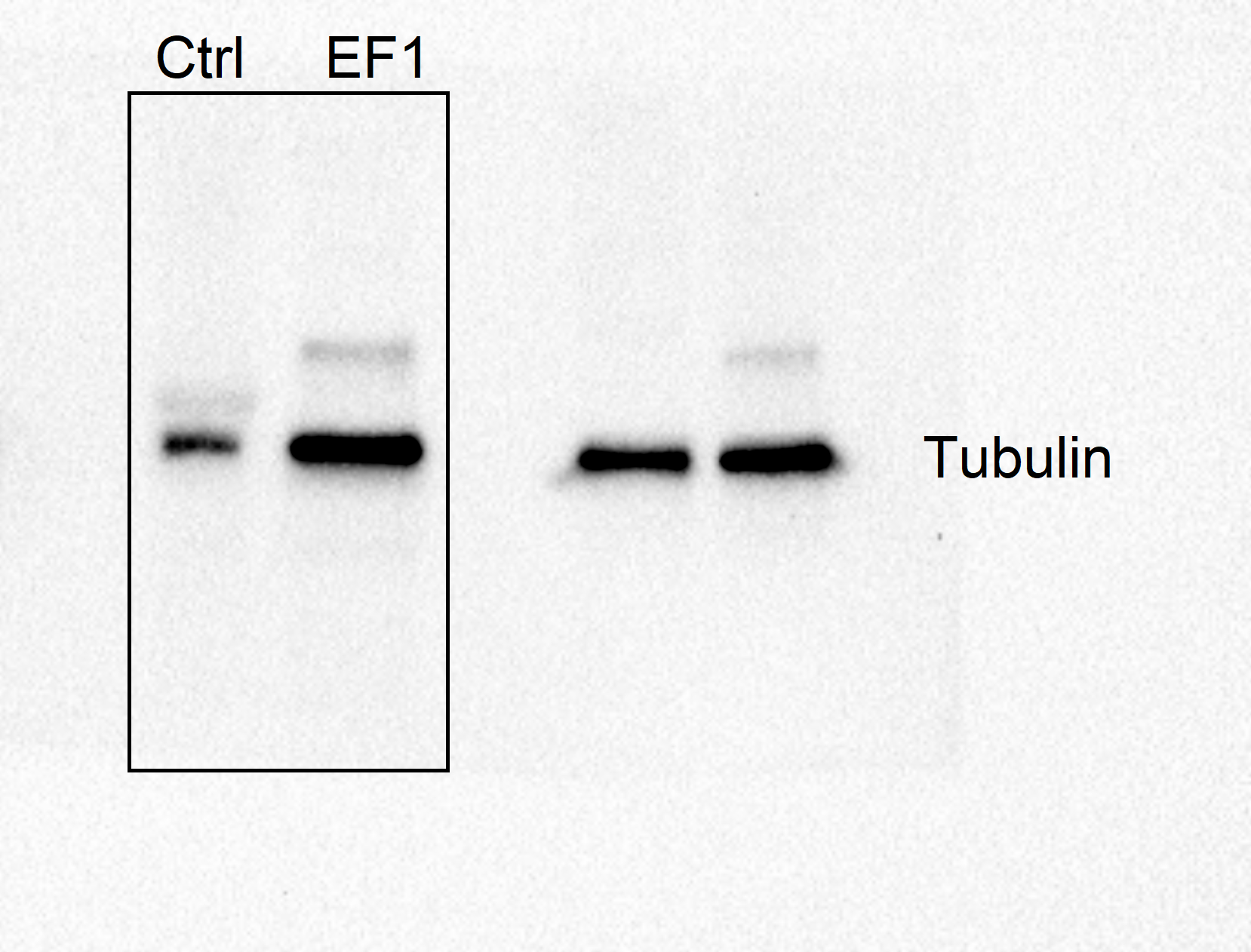

Supplement: Source data 1. [file elife-69734-data1.zip › Vasileva_Source Data/Figure 2-source data 8.tif]

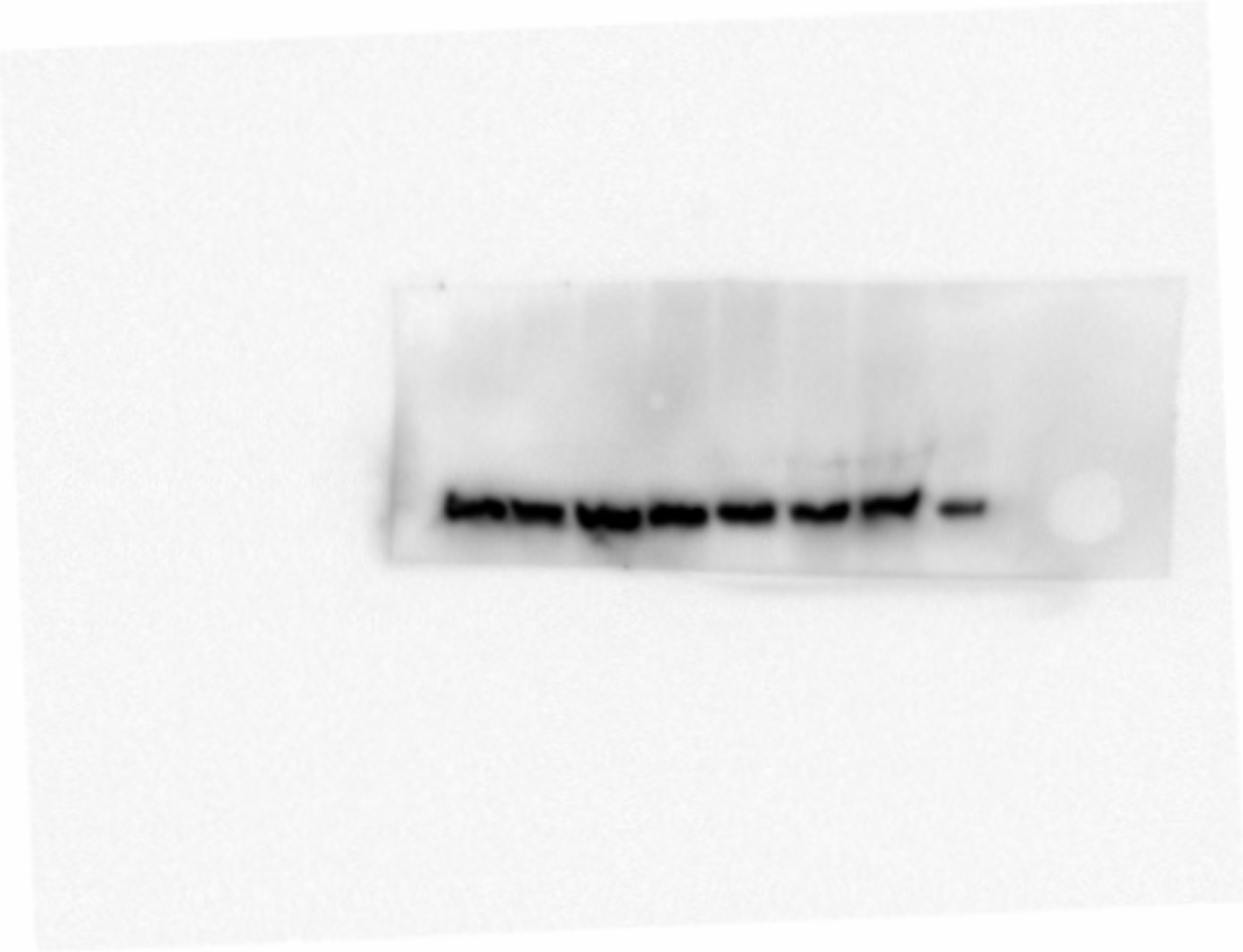

Supplement: Source data 1. [file elife-69734-data1.zip › Vasileva_Source Data/Figure 3-source data 3]

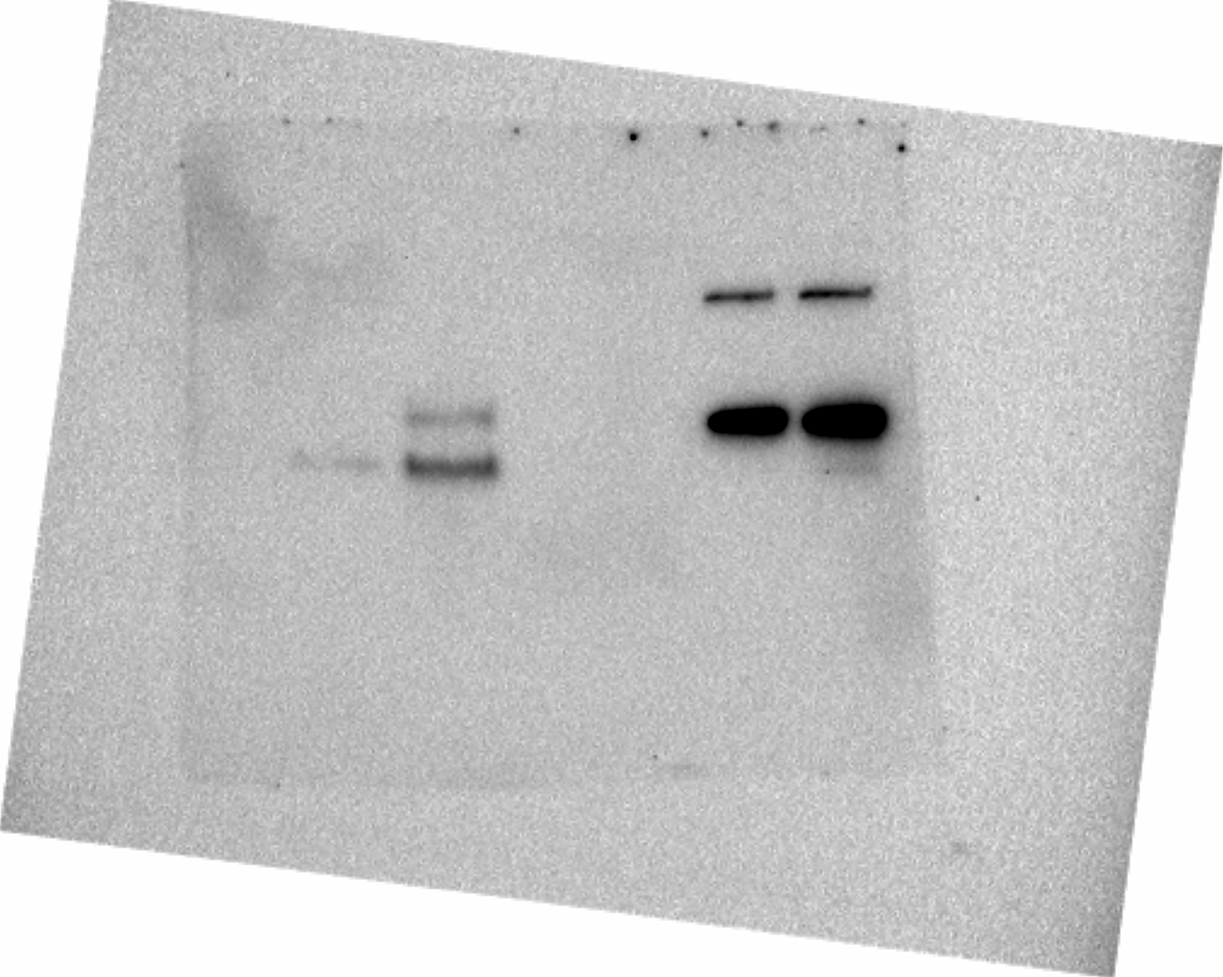

Supplement: Source data 1. [file elife-69734-data1.zip › Vasileva_Source Data/Figure 1-figure supplement 2-source data 1.tif]

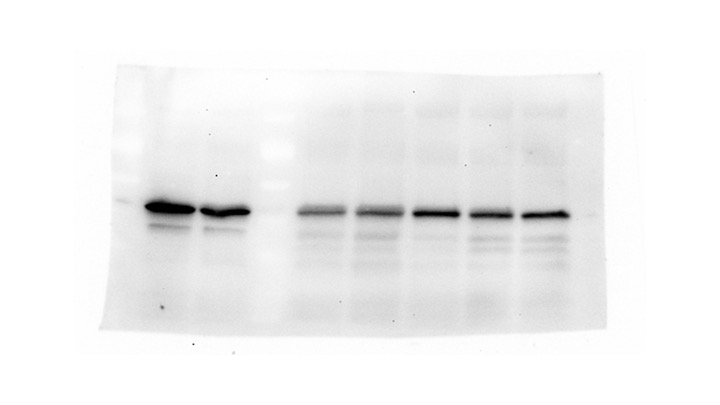

Supplement: Source data 1. [file elife-69734-data1.zip › Vasileva_Source Data/Figure 7-figure supplement 1-source data 3.jpeg]
